# Menstrual Hygiene Management and Waste Disposal in Low and Middle Income Countries—A Review of the Literature

**DOI:** 10.3390/ijerph15112562

**Published:** 2018-11-15

**Authors:** Myles F. Elledge, Arundati Muralidharan, Alison Parker, Kristin T. Ravndal, Mariam Siddiqui, Anju P. Toolaram, Katherine P. Woodward

**Affiliations:** 1 Health and the Environment, Biomass Controls, Durham, NC 27701, USA; 2 Water Aid India, New Delhi 110029, India; arundatimuralidharan@wateraid.org; 3 Cranfield Water Science Institute, School of Water, Energy and Environment, Cranfield University, Cranfield MK43 0AL, UK; a.parker@cranfield.ac.uk (A.P.); kristin.t.ravndal@cranfield.ac.uk (K.T.R.); anju.p.toolaram@cranfield.ac.uk (A.P.T.); 4 RTI International India, New Delhi 110037, India; msiddiqui@rti.org; 5 RTI International, Seattle, WA 98104, USA; kwoodward@rti.org

**Keywords:** water and sanitation, gender, menstrual hygiene management, menstrual hygiene waste disposal, environmental health, sanitary waste

## Abstract

Menstrual hygiene management (MHM) has gained some attention and several literature reviews have been published. However, both original papers and reviews tend to focus on absorbent access and use and not on the disposal of menstrual waste. This review aims to fill a gap in the water, sanitation and hygiene (WASH) sector by bringing a focus specifically on menstrual hygiene safe disposal in low- and middle-income countries (LMIC). We reviewed published literature since 2002 on menstrual hygiene with a focus on menstrual waste management and menstrual absorbent disposal in LMIC. Database searches were conducted of both peer reviewed literature and grey literature, in addition to hand searching of references of relevant earlier literature reviews. In total 152 articles and reports were identified and 75 met the inclusion criteria and was included in the final review. Existing polices on MHM was also reviewed with a focus on India and South Africa. The review showed that disposal of menstrual waste is often neglected MHM and sanitation value chains, leading to improper disposal and negative impacts on users, the sanitation systems and the environment. Findings call for further research to gain better understandings of MHM waste streams, disposal behaviors, absorbent materials and waste management technologies to deliver health, safety, mobility and dignity for women and girls.

## 1. Introduction

Women and men have specific sanitation needs, preferences, access requirements, and utilization patterns and experiences [1]. Women also use toilet facilities to manage their menstruation. Good menstrual hygiene practices means that women and adolescent girls are using a clean menstrual management material to absorb or collect menstrual blood, that can be changed in privacy as often as necessary for the duration of a menstrual period, using soap and water for washing the body as required, and having access to safe and convenient facilities to dispose of used menstrual management materials [2]. Poor menstrual hygiene management (MHM) can negatively impact the health and psycho-social well-being of women and girls [3,4,5]. Menstrual hygiene management in the water and sanitation sector is not formally defined in the Sustainable Development Goals (SDGs). However, clear linkages are framed here to include: SDG3 (physical health and psycho-social well-being for women and girls), SDG4 (quality education for girls), SDG5 (gender empowerment and equality), SDG6 (water and sanitation), and SDG12 (responsible consumption and production for the environment).

A woman menstruates between puberty (age 11–24) and menopause (age 45–55) for an estimated 459 cycles during her lifetime [6]. With rapid urbanization, rising incomes, expanded product availability and distribution, and increased mobility, the use of disposable sanitary napkins is increasing rapidly [7]. A PATH study estimated that the annual solid waste load of disposable sanitary napkins was higher than any other menstrual hygiene product—i.e., 44,254 cm^3^/female/year [8]. Shared and public facility maintenance is frequently a source of environmental health risks due to poor hygiene. In countries where there are many stigmas and taboos around menstruation, poor waste management on-site creates anxiety and stress. This facility maintenance concern combined with the fact that urban waste collection systems are problematic in many low- and middle-income countries (LMICs) creates exposure risks and environmental pollution in dense urban areas.

Few articles discuss or define what appropriate or safe disposal and management of menstrual waste entails. An underlying reason could be the lack of clarity and consensus over how menstrual waste is classified (for instance, as solid waste, hazardous waste, or bio-medical waste), which makes it difficult to offer clear guidance on how best to discard used products, leading to inappropriate and unsafe disposal practices [9] House [4] notes that menstrual hygiene friendly infrastructure includes “clear mechanisms for collecting and disposing of menstrual waste,” but does not elucidate what these mechanisms could be. Incorporating MHM considerations to include waste management into WASH sector planning will advance goals to ensure safety, dignity, and deliver demand-generated designs for women and girls by responding comprehensively to their biological needs [10]. Better MHM is important for sanitation access, sustained facility use, and for gender equity. Improved access to culturally acceptable MHM in sanitation facilities enables women and girls to fully engage in education and at the workforce [11].

Toilet facilities designed to accommodate menstrual hygiene practices, provide access to absorbents, and encourage safe handling and disposal of used absorbents are important measures supporting women’s health and dignity. In the design of communal toilets, disposal of menstrual waste has often been overlooked, leading to improper waste disposal [8,12]. To improve this situation in the future, knowledge of the variety of current menstrual materials is required. For example, if thermal treatment technologies are considered, these will need to be optimized for different materials. Disposing of menstrual blood can have particular cultural considerations, and the breadth of these needs to be fully understood before designing a technology.

This paper examines peer-reviewed and grey literature about menstrual waste disposal, with the aim to inform water, sanitation, and hygiene (WASH) programming on better facility user designs and waste management practices to support the menstrual hygiene needs of women and girls, and the environment. The paper does not attempt to review the broad topic of municipal solid waste management, but maintains a narrow focus on menstrual hygiene waste and waste management. This paper addresses topics of menstrual absorbent use, and brings a unique focus on disposal practices, waste treatment strategies such as incineration, the health and environmental risks associated with disposal, and policy guidance on menstrual waste management. The focus on urban populations is prioritized given the higher public and environmental risks associated with poor waste management in heavily used shared spaces in densely populated areas. From a policy perspective, India and South Africa were a primary focus given these countries large sanitation needs, and their emerging strong commitment to addressing sanitation conditions.

## 2. Methodology

A structured search was performed to identify journal articles, reports, and other grey literature related to menstrual hygiene management and disposal of absorbents. Grey literature was included in the search due to the anticipated limited amount of peer-reviewed literature available specifically on menstrual waste disposal. The search took place in October through November of 2017 and included literature published in the past 15 years (since 2002). The primary focus of the search was on disposal and management of menstrual waste. Specific topics included: types of absorbents used and disposal practices in urban and peri-urban areas of developing countries; socio-economic factors influencing disposal practices; safe disposal’s influence on empowerment and dignity, as well as sanitation practices; environmental and public health risks from menstrual waste; health and environmental risks from incineration; cultural factors related to use and acceptance of incineration; disposal practices in public and institutional settings in the developing world; and policies or guidelines for menstrual waste disposal.

### 2.1. Search Strategy

A database full text search was performed to identify relevant peer-reviewed journal articles. Databases searched included PubMed, Web of Science, Google Scholar, EMBASE, Conference Papers Index, and Scopus. New York Academy of Medicine Grey Literature Database (NYAM or greylit.org) was also included in the database search. An environmental scan for additional grey literature was performed using Google.com, and by directly searching targeted websites of relevant organizations to identify additional reports not already identified through the database and Google searches (e.g., Cranfield MSc thesis database, WEDC database, and organization websites such as PATH and WaterAid).

Key words used to create database search strings included: Menstrual hygiene management, Menstrual hygiene waste management, Menstrual waste management, Menstrual hygiene management disposal, Menstrual waste disposal, Menstrual hygiene management disposal practice*, Menstrual waste disposal practice*, Menstrual hygiene, management disposal technolog*, Menstrual, waste disposal technolog*, Menstrual hygiene management incineration, Menstrual waste incineration, Menstrual hygiene management absorbent, Menstrual absorbent, Menstrual hygiene management health, Menstrual hygiene management risk. The search strategies included a combination of these keywords, including relevant database specific subject terms such as Medical Subject Headings (MeSH) in PubMed. An example search string included: (menstrual OR menstruation) AND (waste* OR hygiene) AND (manag* OR dispos* OR disposal OR “disposal practice*” OR “disposal technolog*” OR incinerat* OR burn* OR absorbent* OR superabsorbent* OR health* OR risk*). The bibliographies of relevant review articles identified through the database search were hand searched to identify additional relevant articles for inclusion.

**Figure 1:**
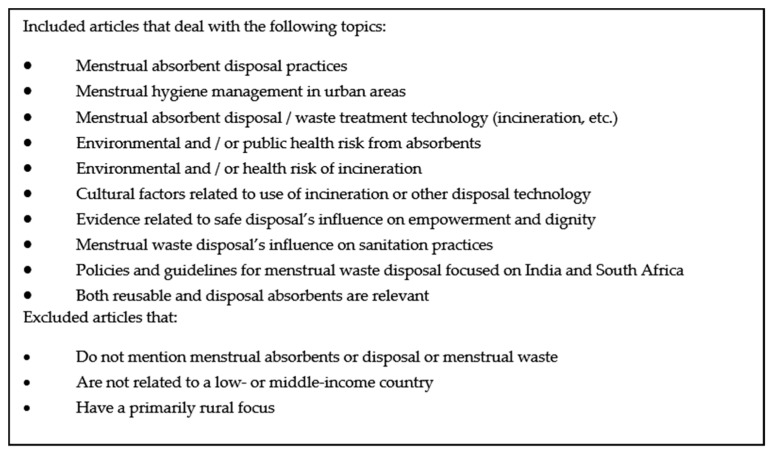
Inclusion/Exclusion Criteria for Literature Review.

Literature from the searches was compiled and 152 article abstracts were screened for initial inclusion using the screening criteria outlined below in Figure 1. A set of full text articles and reports were then reviewed for literature meeting the inclusion criteria after abstract review. In cases of uncertainty, a second person reviewed the article and a decision was jointly made on inclusion.

### 2.2. Inclusion and Exclusion Criteria

The search was global, with a focus on low- and middle-income countries. For the database search, only full text articles published in English since 2002 were included. Letters to editor, op-eds, and editorials were excluded. For the grey literature search using Google, reports, reviews and other articles were included. Presentations, op-ed pieces, and letters to editor were excluded. Inclusion/exclusion criteria are shown in Figure 1.

### 2.3. Search Results

**Figure 2:**
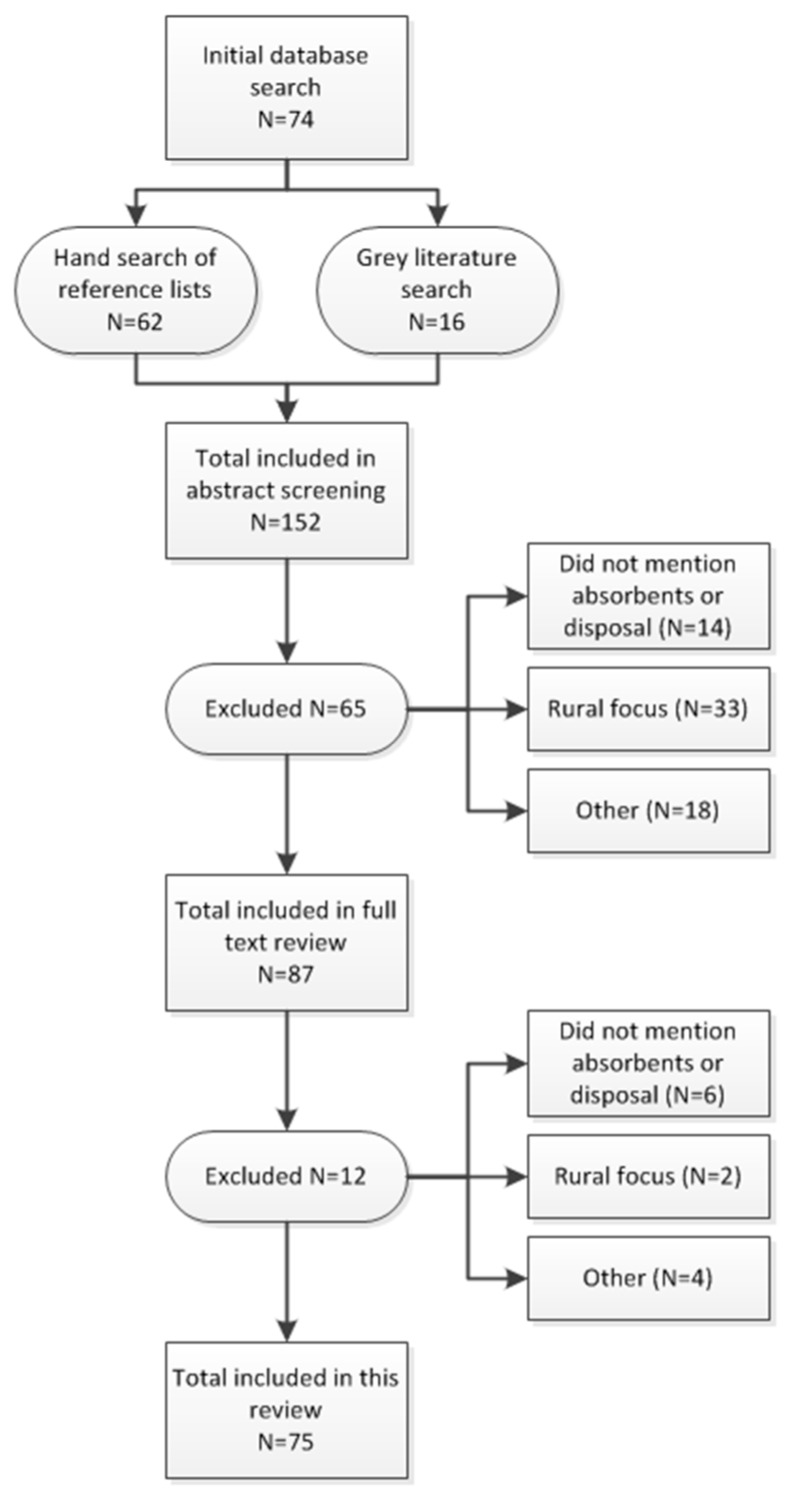
Literature Search Process Flow.

The search process flow and results are summarized in Figure 2. The database search returned 74 articles. An additional 16 articles were identified through grey literature searches, and 62 articles were identified through hand searches of review article reference lists, for a total of 152 articles. After abstract screening, 65 articles were excluded from further review for reasons of not mentioning menstrual absorbents or disposal (14 articles), having primarily a rural focus (33 articles), and other reasons such as a focus on product development and safety, a focus on disasters or emergencies, acceptance studies or intervention studies for a specific type of absorbent, and training materials (18 articles). After full text review, another 12 articles were excluded for reasons of not mentioning menstrual absorbents or disposal (6 articles), having a primarily rural focus (2 articles), and other reasons such as not providing data or new information, not disaggregating urban and rural data, and describing a product but not the use or behavior associated with it (4 articles). A total of 75 articles were included in the final review.

Articles were categorized into relevant topic areas and are discussed according to these topics in this review paper. Topic areas include:

- MHM waste management and disposal practices (50)
- Incineration (15) and public or institutional settings (33)
- Types of absorbents used (53)
- Environmental, public health, or women’s health risk of menstrual waste (18)
- Safe disposal’s influence on empowerment, dignity, or sanitation practices (10)
- Policies and guidelines for menstrual waste disposal (12)

Review findings for of these topic areas are described in the following sections.

## 3. Types of Absorbents Used

**Figure 3:**
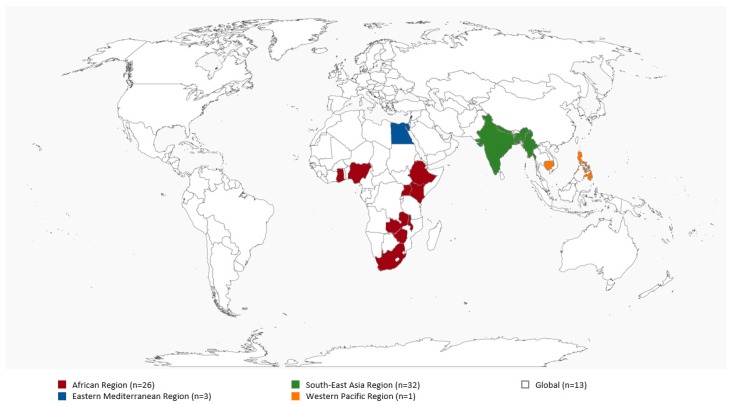
LMICs Country & Regions for Articles Reviewed on Menstrual Absorbents.

At the core of MHM is the use of menstrual hygiene products, a component intimately linked with disposal preferences and practices. These materials are required to absorb or collect menstrual blood safely, comfortably and discreetly. This literature review revealed no universally acceptable classification or typology of menstrual hygiene products; categorization can be according to type, quality characteristics or hygiene parameters. Thus, for the purpose of this review, we have grouped menstrual hygiene products into sanitary napkins of commercial products such as disposable sanitary pads or low cost such as locally made reusable pads; traditional absorbents such as cloth, clothing, cotton wool, toilet paper; and/or unconventional commercial products such as tampons and menstrual cups (Table S1). Supplemental details on the literature reviewed on menstrual absorbents is outlined in Table S2, and Figure 3 maps studies by country. The quality of the menstrual hygiene product is judged by factors like leak protection, absorbency capacity, dryness, product comfort and size, thinness, allergies and biodegradability [13]. Some authors defined menstrual hygiene products either as hygienic (e.g., sanitary pads) or as unhygienic absorbents (e.g., cloth, tissue papers and cotton wool) based on whether their use may cause infections [3,14,15,16]. However, Sumpter and Torondel [3] state that the hygienic use of absorbents, whereby they are washed and dried properly, is what is critical.

Studies across the urban areas of LMIC have reported sanitary pads, cloth and tissue paper as the most commonly used menstrual hygiene products [3,17,18,19,20]. Additionally, products/absorbents such as homemade pads, clothes, underwear, sponges and cotton wool were also cited [3,17,18,19,20] Table S1 summarizes findings on the type of menstrual absorbents used in LMICS, and while these studies are based on small samples, they reveal important trends in absorbent use. Studies from African countries such as Ghana [21], Nigeria [15,22,23] and Egypt [24,25] indicated that girls in secondary school mainly used sanitary pads. Schoolgirls from Malawi [26] and Ethiopia [13,27] primarily relied on cloth or homemade pads. Asian countries showed greater use of cloth or clothes during menstruation (Table S1). In particular, Anand et al. [16] studied census data (2007–2008) of India and presented the use of cloth as the main menstrual hygiene product used by females aged 15–49 years. However, urban India saw greater use of sanitary pads, a trend likely to increase in the future. [7] Studies done in Nigeria [14,15], South Africa [28], Uganda [29] and in India [7] reported few tampon users. Trial studies for menstrual cups were conducted in Zimbabwe [30] and South Africa [28] with positive feedback; yet the use of insertable products such as tampons and menstrual cups may be hampered by cultural beliefs around virginity and fertility [1,7,29,31] Cloth pads may be traditional, yet the disposable sanitary pad is aspirational for users as it can offer protection against leakage and odor while being comfortable and safe, though it is not always affordable [20,26,29,32] While sanitary pads offer comfortable and leak-proof protection, the composition of pads (cellulose, super absorbent polymers, plastic) has implications for disposal (Table S1) [13,17,20,26,31,32].

Affordability of pads, socio-cultural norms, knowledge, and variation in menstrual flow shaped women and girls’ use of both pads and cloth or other traditional material (Table S1) [7,14,15,17,19,25,29,33,34,35]. Among secondary school girls in Zagazig City, Egypt, 44% disclosed that they used sanitary pads for the first two days of their cycle when their menstrual flow was heavier and switched to cloth for the last few days [25]. Girls in two studies in India preferred to use sanitary pads at school and cloth at home [35,36]. Women and girls may change the type of absorbents they use over time. Women in a study in Durban, South Africa have noted using cloth when they were younger but used sanitary pads when older [37].

Several studies found statistical associations between hygienic practices (at least the use of sanitary pads) and factors such as age, type of schooling, education of mother/father, residential status, social class, economic status, occupation of parents, mass media exposure, as well as knowledge and training (Table S1). In an urban resettlement area of New Delhi, the use of sanitary pads were more likely to be associated with young women (20–29 years old) rather than older women (≥30 years old) and with those whose mothers were better educated, as they provide more information to the girls [38]. Among secondary school girls in Mansoura, Egypt sanitary pads were associated with higher social class, urban residence, and exposure to mass media [24]. Some studies reported that students in private schools used sanitary pads more than their counterparts in government schools [15,39], possibly because the former may be from wealthier families [20].

Although, this review found studies where the authors alluded to these socio-economic and socio-cultural factors associating with the hygiene practices within their study sample, it also revealed that this association sometimes lacks quantitative evidence relying on qualitative and descriptive reasoning (Table S3). Thus, this review highlights the paucity of detailed and controlled studies exploring these relationships that can contribute towards more generalized conclusions to feed into intervention programs and public policies. Similarly to Kuhlmann and colleagues, this review also emphasizes clusters of studies on MHM in Sub-Saharan Africa and South Asia with less studies in other low income regions of the world [20]. Thus, revealing a need for widening global research efforts on MHM since women and girls’ menstrual absorbent preferences and usage patterns are diverse, with possible influences based on location, activity, age, culture, and socio-economic status. Understanding practices and preferences for absorbent disposal is an essential part of the menstrual hygiene value chain.

## 4. Menstrual Waste Disposal Practices

Against the backdrop of menstrual hygiene product use, this review examined how used products are discarded and the factors affecting disposal practices. Women and girls face constraints during menstruation that determines how and where they dispose of menstrual absorbents.

Sommer et al. [17] and Kjellen et al. [12] provide a framework for understanding the disposal of menstrual waste in the context of sanitation systems, calling attention to the various interaction points across the sanitation value chain from the toilet, to waste collection, conveyance, treatment and disposal of excreta.

Table S4 shows the wide variety of ways that women and girls currently dispose of used menstrual absorbents, including throwing them in the open and in latrines, burning, or burying them, and through routine waste disposal systems, with disposal practices often influenced by deeply embedded socio-cultural norms and taboos related to menstruation and menstrual blood. Findings from a systematic review and meta-analysis study in India suggest that unsafe disposal practices such as throwing absorbents in open spaces and burning (likely open burning, not incineration) was significantly higher in community-based studies (especially in rural and slum settings) than in school-based studies, and that reliable solid waste disposal was more common in urban settings than in rural settings [7]. These results suggest that menstrual waste disposal may be more challenging in community settings than in institutional settings because the facilities are not as well managed, further highlighting variations in disposal practices within urban areas, with slum-based studies reporting inappropriate disposal more than urban-focused studies per se. Discarding used menstrual absorbents in latrines was noted in school-based studies, particularly when girls lacked access to dustbins. Two studies among schoolgirls in Ethiopia note particularly high rates of disposal in latrines at 69.3% [40] and 77.5% [27]. In low-income communities in Bangladesh, some women disposed of their used cloth in drains and ditches, but others who were uncomfortable with disposing of menstrual cloth in the open threw them in toilets, perceiving that as a discrete disposal option [41].

In a study of communal ablution blocks (CABs) in Durban (South Africa) none of the studied CABs had bins for disposal within the toilets [8]. Dustbins with lids and liners installed inside the toilet cubicles have been successfully tested in communal toilets in a slum in Dhaka, Bangladesh [41], and were a common request by women using CABs participating in the study by PATH [8]. However, in South Africa, women reported that they do not throw used pads in the municipal dustbins as they feared that dogs will dig out the used pads, and someone will see them and think poorly of them [37]. Some used an old paint tin or bucket with a lid held down by a stone in the yard to privately dispose of used pads. Women who did not have any way to discard their pads keep them at home under their bed for days until they found some way of discarding them with other household waste [37]. The type of absorbent may influence how it is disposed. Nair et al. [35] highlight mixed use of cloth and pads among female students in a South Indian city, noting that 76% of girls burned used cloth. While many articles suggest that girls simply discard used materials as is (without any wrapping), a few studies found that girls wrapped used pads in paper or plastic before disposing of them [25,33,37], and this practice is being promoted on some absorbent manufacturers’ packaging or through solid waste segregation strategies.

A few studies noted that poor water, sanitation and hygiene infrastructure, including disposal, made it difficult for girls to manage their menses in school, yet few explicitly recognized the need for disposal facilities as part of WASH or sanitation facilities in schools [17,24,27,32,42,43,44]. In Accra, Ghana, Sommer and Ackatia-Armah [18] noted that schools had insufficient toilets, inadequate privacy measures in toilets, and inadequate disposal facilities for used absorbents. In the Philippines, a UNICEF study reported that toilet stalls rarely had dustbins and that girls were often asked to carry their trash back home [32]. In Malawi, sanitary pad users found disposal “awkward” in the absence of dustbins and incinerators, leading them to keep their used pads/cloth with them, under their bed [26]. In Nepal, 28.2% of girls noted the absence of disposal facilities in schools as a reason for missing school days, especially on days when they had to change their absorbent more [44]. In Ethiopia, 69.3% of schoolgirls reported feeling uncomfortable in school during menstruation primarily because they lacked a private space to change their absorbents (39.2%), did not have access to water for washing (19.1%) and disposal facilities (10%) [40]. Another study in Ethiopia found that 8.5% of girls shared that they remained absent from school during menstruation because of the lack of disposal facilities for used pads and cloth [27]. While there have been several studies of MHM in schools and communal studies, MHM in workplaces is still an under researched topic [10]. In urban areas girls and women often work, travel and live in overcrowded areas lacking privacy and hygienic spaces for MHM, including disposal options [10].

Incineration is an option for managing disposal of menstrual waste, particularly in worksites, schools and dormitory settings. It is a manner to achieve pathogen treatment, waste reduction and on-site waste management. Incinerators properly vented and directly connected to the toilet room by a chute provide an effective and discrete way of disposing of menstrual absorbents [1]. A small number of papers on the use of incinerators in LMIC were identified in this review; this is most likely due a lack of research conducted on the topic. A range of different technologies exist from basic ceramic pots to complex systems with energy recovery, each having advantages and disadvantages (Table S5). A number of factors have to be taken into consideration when selecting what type of incinerator to use, including the types of absorbents used and their composition, where the incinerator should be installed (e.g., household, communal toilet, institutional settings), the volume of waste to be incinerated, appliance treatment temperatures, capacity, emissions, the budget available and the capacity for operation and maintenance. Several examples of the technologies used were found to be less complex incinerator technologies (clay pots, low cost locally made incinerators and electric incinerators). In this review, no examples were found that use high temperature incinerators for bio-medical waste or incinerators with waste to energy technology for managing disposal of menstrual waste in developing countries.

Incinerators have been installed in settings such as households, [45,46] communal toilets, [45] and schools [12,26,47,48,49]. In a study by Elawati [47] on incinerators in schools in Nepal, 46% of girls felt that incinerator use was easy, while 5% were uncomfortable using the incinerator, and 49% had not used the incinerator facilities yet [47]. Socio-cultural beliefs regarding the disposal and burning of menstrual blood could be a reason for why some girls did not discard of used pads in the school incinerator. Girls in one study noted several benefits of having an incinerator in the school including ease of changing pads in schools (34%) and improved school sanitation facilities as a whole (42%), suggesting a clear preference for incinerators in schools to discard menstrual waste as opposed to other methods [47]. During a workshop, schoolgirls from Malawi noted the need for sanitary pads (26%), water (17%), and incinerators (11.4%) to help them manage their menses better [26] While incinerators are considered convenient and facilitate onsite disposal, there also are reports of broken school incinerators not being used [13], problems with smoke and smell from simple incinerators in schools [12], and concerns voiced about emissions released from incinerators [50,51]. Women using a communal toilet in Tamil Nadu were reluctant to leave menstrual waste in communal bins as it was unclear when the waste would be burned [45]. The majority of participants were comfortable with communal incinerators, but the Tamil Nadu study found that incinerators installed in a communal toilet were not in use due to a lack of signage [45]. In some communities, the burning of menstrual blood is taboo, drawing on the belief that such actions will compromise a woman’s reproductive capacity [52]. However, findings from Nigeria ran counter to socio-cultural norms that prohibit the burning of menstrual blood in the country—over half the girls interviewed burned their pads, as they believed this to be the only method that removed all traces of menstrual blood [23]. Privacy has often been overlooked for the design and placement of incinerators [17]. To ensure privacy, incinerators should have a chute directly from the toilet room to the incinerator; this design has been tested in Tamil Nadu [49].

Across the world, girls and women are subject to anxiety and stress with the management of menstrual waste. In many settings, a culture of silence and shame centers on the perception that menstrual blood is dirty or impure [19]. Consequently, girls observe a number of restrictions during menstruation and take measures to hide traces of menstrual blood. The sight of menstrual blood on used absorbents is believed to cause harm to those who see it or come in contact with it, and some fear that it may be used for black magic [45]. In order to remove traces of blood from absorbents, girls may wash used pads [39,52], smear them with mud [52], wrap them in paper or in plastic bags [45], or keep them until they can be discarded discretely [36,37,53]. Managing menstruation in camps (e.g., refugee camps and camps for internally displaced persons) is challenging, and even when dustbins are provided for waste, women may choose more discrete methods for disposal such as burial [54]. The need for discretion may also explain why some throw pads in toilets [40,41].

### 4.1. Behavior Regarding Disposal

Disposable sanitary pads are more available than ever before, underscoring the need for disposal facilities. Several interventions on menstrual hygiene management suggest that health education and hygiene promotion activities in schools increased access to and use of disposable sanitary pads, encouraged frequent changing of pads (2–3 times a day), or recommend disposal of used reusable cloth after a few months of use. Assessments of two interventions found that more girls discarded their cloth reusable absorbents after 3–4 months use as opposed to throwing them away after one a year of use [25,33]. These studies also found that post intervention, more girls changed their cloth and disposable sanitary products more frequently, reporting changing disposal pads 3–4 times a day after the intervention [25,33]. In Nigeria, Aniebue et al. [43] found that girls who received pre-menarche training were more likely to dispose of their used absorbents appropriately than those who had not received training. A critical factor affecting disposal is the disconnect between water, sanitation and hygiene facilities, and disposal, particularly in institutional settings. A few studies noted that poor water, sanitation and hygiene infrastructure, including disposal, made it difficult for girls to manage their menses in school, yet few explicitly recognized the need for disposal facilities a part of WASH or sanitation facilities in schools [17,24,27,32,42,43,44]. In Accra, Ghana, Sommer and Ackatia-Armah [18] noted that schools had insufficient toilets, inadequate privacy measures in toilets, and inadequate disposal facilities for used absorbents and advocate for responsive WASH facilities in schools that can enable girls to manage their menses more hygienically and with dignity.

### 4.2. The Effects of Disposal Practices on the Health and Wellbeing of Girls and Women, and the Environment

Poor menstrual hygiene practices, specifically unhygienic use of menstrual hygiene products, can pose risks to women’s health, such as increased risk of urogenital infections, though scientific evidence of this link is limited and does not provide concrete evidence of an association between poor MHM and adverse health outcomes [3,5,16,55]. A systematic review by Sumpter et al. [3] found that of the 11 studies looking at reproductive tract infections (RTI), seven found an increased risk associated with “worse” MHM (defined differently for each study but generally meaning not using disposable sanitary pads), one found the reverse to be true (an increased risk from using disposable sanitary pads), and three found no association. Sumpter et al. [3] calculated an overall pooled OR of 1.07 (including only high-quality studies with laboratory-confirmed cases), which was not significant. Three additional studies published since the Sumpter et al. (2013) [3] review are included here. A case-control study by Das et al. [5] in Odisha, India found women who used reusable pads were 2.3 times more likely to have symptoms of urogenital infection and 2.8 times more likely to be diagnosed (via laboratory confirmation) with at least one urogenital infection (bacterial vaginosis (BV) or urinary tract infection (UTI)) than women using disposable pads. Controlling for environmental and other confounding factors, the study found that increased wealth and space for managing menstruation were protective for BV. Lower income, poor water access and lack of latrine in the household were associated (though not significantly so) with testing positive for BV (AdjOR = 0.5) and BV/UTI (AdjOR = 0.6) [5]. A cross-sectional study in India by Balamurugan and Bendigeri [55] found similar results: 38% of women who used cloths during menstruation had a laboratory-confirmed RTI such as BV compared to 15% of women who used sanitary pads. A study from Anand et al. [16] in India found that women who used unhygienic methods during menstruation, including anything other than sanitary napkins or locally made napkins, were 1.04 times more likely to report any symptom of RTI and 1.3 times more likely to report abnormal vaginal discharge than women who used hygienic methods, such as sanitary napkins. In rural India, there were statistically significant associations between type of menstrual absorbent used and absenteeism among adolescent girls during menstruation (OR 1.43, 95% CI: 1.04–1.97; *p* < 0.001) due to inadequate disposal facilities at school [56].

A few studies in this review discussed the interactions between menstrual waste and sanitation systems, noting that the disposal of used menstrual absorbent products in toilets is challenging for both on site sanitation facilities and sewerage systems, particularly in urban areas [8,12,17]. Two attributes of menstrual absorbents affect disposal with implications on the sanitation system: the type of material (whether biodegradable, non-biodegradable, or mixed materials) and the type of use (single use or reusable) [8,37]. Sewerage systems are designed to carry water and fecal matter; discarding solid waste such as pads and cloth in toilets can block sewage pipes, pose challenges for wastewater treatment plants, and can have significant cost and human resource and dignity implications. Onsite sanitation facilities such as pit latrines are constructed to aid the breakdown of organic material. The disposal of non-biodegradable pads and cloth made of synthetic materials runs counter to this process, compromising the aerobic and anaerobic digestion processes that break down fecal matter in the leach pit [12]. Further, when used absorbents are wrapped in plastic and disposed, it can slow down if not disrupt the decomposition of biodegradable components of the pad [12]. In the case of septic tanks, sanitary pads make emptying and cleaning challenging and cumbersome [8]. Disposable sanitary pads made with super absorbent polymers (SAP) are particularly challenging as SAP absorbs fluids, causing the pad to swell up and block sewer lines [17]. This has implications on the health and social status of sanitation workers and cleaners who have to remove blockages from sewers and clean septic tanks [8,17]. In Dar es Salaam, Tanzania, there are an average of 150 sewer blockages per month costing the Dar es Salaam Water and Sewerage Corporation US$25,000; menstrual waste is a common reason for this [12,17]. Similarly, in Eastern Kenya, the Mavoko Water and Sewerage Company reported that menstrual pads constitute about 40 percent of the material cleared from blocked sewers [12,17]. The eThekwini Water and Sanitation utility in Durban, South Africa, reports frequent blockages of the suction hoses used for pit emptying due in large part to presence of menstrual materials in the latrines [12,17].

Current solid waste management laws in India require the waste generator to securely wrap sanitary waste prior to disposal and require the product manufacturer to provide a wrapper for disposal. To address these requirements, manufacturers have started providing plastic wrappers for product disposal, but an unintended consequence of this is the increased burden on the environment and waste management systems from the additional menstrual waste wrapping [50]. The Government of India is promoting incineration of menstrual waste as a disposal method, which would help to reduce environmental burden of menstrual waste if incinerators meet design and emission standards. However, the lack of standards for small-scale appliances, the lack of oversight or enforcement of emission regulations on incinerators, the potential risks resulting from inadequate emission control measures or poor thermal treatment performance are concerning [51,57].

A potential health concern arising from incineration of menstrual waste is that certain components of menstrual products may become toxic upon burning. For example, some versions of sanitary napkins often contain chlorine bleach (to appear white in color), which emits dioxins, a human carcinogen with developmental and reproductive effects [58], when burned at a low temperature [17]. Release of dioxins and other toxins is minimized when menstrual waste is burned at a sufficiently high temperature to ensure complete and efficient combustion [17]. For bio-medical health waste, the WHO recommends that small-scale incinerators reach a temperature of at least 850 °C according to EU and South African standards or 1000 °C based on Indian and Thai standards [59]. The European Waste Incineration Directive recommends incinerators reach a temperature of at least 850 °C for at least two seconds to ensure full breakdown of toxic substances [60]. Specific guidelines on the incineration of sanitary pads are lacking, and the same standards applied to medical waste may not be appropriate. Additional research and better guidance are needed for stand-alone small-scale incinerators as many of these guidelines are geared to larger appliances than are commonly used in institutional settings or are mainly addressing medical waste and not menstrual waste. More research is needed to better understand the risks posed to health and the environment from onsite incineration of menstrual waste [17].

## 5. Policy Considerations

Clear-cut policy and guidelines for menstrual hygiene management, menstrual hygiene products and waste disposal are quite limited in the review articles. Most articles reviewed in this search focus on barriers and challenges experienced, yet few provide references to directives or operational guidance on gender-informed interventions [1], good MHM practices or safe and hygienic absorbent use, or policies governing disposal options and waste management practices in various settings, including worksites [10].

Literature on the policy elements of menstrual hygiene management in WASH, and especially on the topic of waste disposal and management is scarce. While a number of countries, such as India, Ethiopia, Kenya, South Africa, and Uganda, are putting in place better policies for menstrual hygiene, few articles examine the impact of these policies on programs and practice particularly on the waste management dimension.

A policy analysis for school WASH and MHM in India noted that MHM is factored into school guidance, though often without implementation details or accounting for site or cultural nuances [61]. This lack of clear administrative responsibility extends to work sites as well; little guidance exists to address the MHM needs of women and girls in their place of work [10]. Given the problems of disposal in latrines and sanitation systems in terms of operations and maintenance (O&M) [1,4,8,12,17], the O&M dimension is lacking in policy and guidelines for WASH facility management. India’s policy directive on MHM have progressed since 2015, with National MHM Guidelines for schools released in December 2015, and a draft resource document on menstrual waste management for rural areas available as of 2018.

There is ambiguity around government stakeholder’s roles for sanitation in the context of MHM policy and guidelines which may influence the level of attention to disposal. The health sector has focused on public health education and absorbent access, the education sector on gender-segregated toilets for schools and some health education, and other agencies on constructing rural or urban sanitation facilities [48], highlighting a striking gap where no one agency gives full attention to all dimensions of MHM to thoroughly address disposal practices and facilities.

Incinerators are a population waste-management solution in worksites and educational settings in LMICs like India, yet global guidance or national policy to address thermal treatment of sanitary pads is not clearly available. Where facilities for disposal are provided there are examples of successful [47] and not so successful user adoption or quality facility O&M [12,17,50]. Directives on incineration procurement as per quality standards, installation and operation are difficult to find from appliance manufacturers, or from governments and schools that may deploy the technology. Guidance on performance criteria and quality standards, the use of incinerators at-sites, and greater recognition of cultural perceptions, stigma and taboos associated with menstrual disposal and the use of on-site incinerators [12,62] could contribute to supportive use cases.

## 6. Discussion

Menstrual hygiene management in the water and sanitation can be linked to and supportive of the Sustainable Development Goals (SDGs), including SDG 3 (physical health and psycho-social well-being), 4 (quality education), 5 (gender empowerment and equality), 6 (water and sanitation), 11 (sustainable cities), and 12 (responsible consumption and production for the environment). As is the case with MHM programs, addressing menstrual waste management calls for an inter-sectoral approach across drawing upon the roles and strengths of the health, education, and sanitation sectors (both government and non-government).

As we see more nuanced and responsive MHM and sanitation policies and programs in LMICs, the findings of this review suggest important considerations related to absorbent use, waste disposal guidance and practices, and the effect of disposal on the health and the environment that must be integrated.

Menstrual hygiene product availability and use: The range of available menstrual hygiene products, both disposable and reusable, creates opportunity for users to make choices in line with their physical and contextual needs, and cultural and socio-economic circumstances (refer Table S1). Disposable pads are of different types, some with added characteristics (e.g., scented, ultra slim, superabsorbent, with added medicinal properties), and are favored in many settings for their convenience and reliability. This sheer variety of disposable pads makes MHM a dynamic issue with the diversity of menstrual waste being generated (refer Table S1). Greater knowledge of product availability, use and disposal patterns will help facility planners and policy makers anticipate menstrual waste streams and design appropriate washing and toilet spaces to accommodate those waste streams and volumes.

New product developments and trials on reusable products (different types of pads made of cloth and other materials, menstrual underwear, menstrual cups), and compostable disposable pads offer the potential for reduced waste streams. However, standards and regulations for these products do not exist in many countries, necessitating performance research to define quality standards to guide future product development. The establishment of performance and quality standards for such products is critical for hygienic use, and has important implications for disposal and waste management.

**Figure 4:**
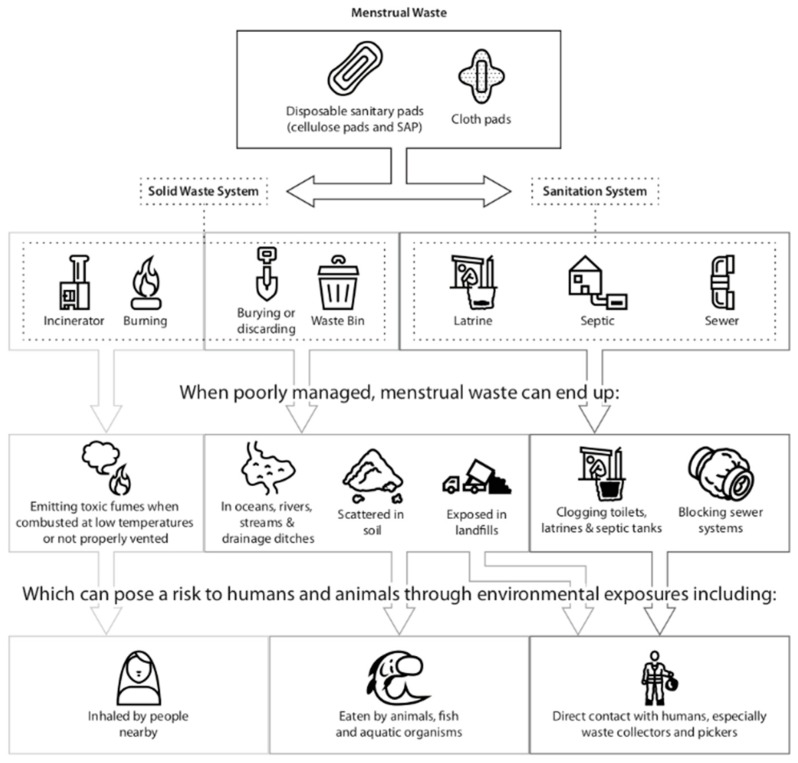
Waste disposal pathways and potential environmental and health hazards.

Menstrual waste guidance and practices: The review shows that menstrual waste management is largely neglected in MHM and sanitation value chains, resulting in unguided and inappropriate management at individual, community and institutional levels [8,9,12,17]. At present, menstrual waste is discarded in safe and unsafe ways, posing difficulties to the user, sanitation systems and the environment. There are individual case studies of the impact of inappropriate disposal on both on-site and sewerage systems, though this does not yet translate into any documented impact analysis, or broad changes in practice. Figure 4 depicts the commonly observed disposal pathways drawing attention to the implications of poor waste management on users, sanitation workers and the environment. This figure further suggests the need for software (e.g., awareness, capacity building), hardware (e.g., technology), and governance interventions at various levels of the waste management value chain from the discarding used materials at the user level, the collection and management of accumulated waste, and final treatment and disposal. Figure 4 depicts the commonly observed disposal pathways.

A central issue that underlies the governance or implementation of menstrual waste management is how country governments categorize menstrual waste—it could be common household waste, hazardous household waste (required to be segregated from routine household waste), biomedical waste given the absorbed blood content, or plastic waste given the plastic content in many commercial disposal pads. While the Indian government, under their Solid Waste Rules 2016, classifies sanitary pads as solid waste, policy guidance on sanitary pad waste collection, handling, storage, transportation, treatment through incineration or other methods need detailing. Clear categorization of waste streams may be useful to direct public services for waste collection, or technology choices in institutional settings for the recommendation of incineration, autoclave or microwave use to destroy pathogens in menstrual waste. Furthermore, environmental standards and guidance need to address the fact that commercial tampons and sanitary pads often contain chlorine and polyethene that may produce dioxins and other potentially dangerous chemicals that could contribute to health concerns from air emissions from combustion processes, or groundwater contamination as a result of leaching from unlined landfills and sites where solid waste accumulates.

### Effects of Disposal on Health and the Environment

The review looked for data linking inadequate waste management solutions and women’s health, driven by whether the lack of disposal facilities affects how women use menstrual absorbents. Studies investigating the link between MHM and women’s health outcomes largely rely on self-reported outcomes (e.g., Anand et al. [16]) with a focus on absorbent (mostly cloth) use and not disposal practices. Other more robust clinical studies are often cross-sectional and also focus on absorbent use (e.g., Das et al. [5]), and thus cannot establish a causal link between menstrual hygiene practices, particularly product use (including duration of use) and health outcomes. Public health data on the health risks to sanitation workers and environmental health risks associated with menstrual waste products and their pathogen load is not available in the reviewed literature. Despite these limitations in the literature, review findings do allude to notion that it is not just the type of absorbent used that affects women’s hygiene and health during menstruation, but how it they are used, and facility factors such as having a private space, water, and soap to manage menstruation. The use of laboratory-confirmed cases is a substantial improvement over self-reporting of symptoms, and should include analysis that will help to establish the evidence base related to product use, duration of use, and hygienic management.

The tradeoff between reducing environmental waste against potential health risks to girls and women must be carefully considered when promoting waste management solutions. From a women’s health perspective, a variety of reusable or disposal pads may be safe if used well. The promotion of disposable sanitary napkins versus reusable absorbents may be warranted given the research showing increased incidence of urogenital infection in women using reusable pads, regardless of washing, drying and storing practices [5], though more research is needed. From an environmental perspective, reusable pads are more environmentally friendly, though they require access to clean water and soap for proper washing, and drying facilities. Access to water and space for drying is a real constraint in many poor urban settings which may increase risks for women in some settings. Performance and quality standards for reusable menstrual products and compostable pads need to be established to make these products a viable option for women in terms of their health and from a waste reduction perspective.

Waste from disposable sanitary pads may be treated and waste loads reduced with the use of incinerators. Incineration requires careful management of appliances to ensure complete combustion and pathogen kill, safe operational temperatures, safe installation and controlled emissions or there will be human environmental risks. Incineration is an approach found in many settings for managing menstrual waste in institutional and shared public settings, though it has its own environmental risks and cultural considerations. Incineration may not be acceptable for all jurisdictions and cultures. Decentralized stand-alone incinerators on the market also provide little data on types of pads to be burned, the temperatures or air flow that is prescribed for operation, or the expected emission controls and emission levels. Incineration is an approach that aligns with decentralized waste treatment approaches and systems, and may be an attractive solution for waste management particularly for public toilet settings in communities where solid waste collection systems are weak and in institutional settings (schools and worksites). However, simple incineration appliances may combust at low temperatures, producing toxic emissions, and may be ineffective in killing pathogens. And on-location, incineration appliances may not be installed properly to vent or exhaust air emissions outside of the toilet block. Further, users and operators may not be trained or provided with adequate information to safely operate the technology.

Engineers designing an incineration technology, and policy makers guiding the procurement and installation of such technology need to know what menstrual products are used in order to design efficient thermal treatment products.

## 7. Recommendations

The disposal of used absorbents cannot be understood and carried out in isolation, requiring user-centered design thinking to consider socio-cultural norms regarding menstruation, product availability, quality and use, and existing sanitation systems. In institutional and community settings, the design of sanitation systems needs to be responsive to and plan for disposal of menstrual waste, providing appropriate, discrete and safe solutions that minimize adverse health impacts on girls and women, sanitation workers, and broader environmental consequences.

Our recommendations are framed around three levels of action: research, programs and policy.

### 7.1. Research to Inform Policy, Practice and Technology Development

- Public and environmental health research confirming risks of pathogen transmission from waste products disposed in the open or handled by waste or sanitation workers
- Product research on composition and quality of disposable, compostable and reusable menstrual products, and health effects of these products linked with additives and hygienic use (especially duration of use)
- Research to estimate the health and environmental effects of waste management strategies (e.g., incineration, composting) on different types of products (e.g., emissions from incinerating different types of sanitary pads)
- Further research and development on thermal treatment strategies and technology, examining incinerator types being used, their installation and maintenance, temperatures of operation, and emissions from incinerators
- In-country or country-specific operational research on use of incinerators for menstrual waste management in public toilets and institutional settings (particularly schools and workplaces where installations are most common)
- Policy research on governments’ positioning of menstrual waste management, procurement processes and budgetary allocations for menstrual waste solutions

### 7.2. Comprehensive MHM Programs to Address Product use and Menstrual Waste Disposal Practices in Public Settings

- MHM programs to include attention to informed product choice (whereby girls and women are informed of the range menstrual hygiene products available, their hygienic use, and their advantages and disadvantages), as well as information on and solutions for the safe management of menstrual waste in community, institutional, and public settings. Innovative yet simple behavior strategies to promote sound and appropriate disposal practices to be identified, tested, and scaled up.
- Attention to operations and maintenance of waste management solutions, especially incinerator technologies in different settings, with appropriate and detailed training and guidance to operators and institutions to ensure smooth and efficient functioning of technologies.
- Clear articulation of and operational guidance on menstrual waste management from the time of segregation and disposal by user, to collection, transportation, and final treatment and disposal of waste products.
- Implementation research and monitoring indicators for interventions to include attention to menstrual waste management practices and solutions.
- The unique menstrual hygiene needs of girls and women who are differently abled or in vulnerable situations (e.g., disasters) including their needs and challenges with disposal to be understood and considered within the scope of MHM programs in general, including waste management strategies.
- WASH and MHM sector actor to create global and national platforms for cross-learning and knowledge sharing on the evolving menstrual hygiene product landscape and waste management solutions, engaging industry, government and non-governmental stakeholders.

### 7.3. Policy Advocacy for Safe and Appropriate Management of Menstrual Waste

- Menstrual waste management to be positioned as a critical issue relevant for all government departments addressing MHM in various ways (through education, health, sanitation, women’s empowerment/gender equity), with guidance on how government departments can coordinate to aid comprehensive programming on MHM.
- Government to develop and institute performance standards and regulatory and enforcement mechanisms for menstrual hygiene products and waste management solutions and technologies, and introduce regular monitoring to ensure quality and adherence to standards.
- Adequate budgetary allocations through government policies and schemes for all components of MHM, including menstrual hygiene products and waste management solutions.
- Global guidance documents to include greater attention to MHM, include waste management. For instance, documentation from the World Health Organization (WHO) or UNICEF on core questions and indicators for monitoring WASH in schools and the SDGs should include further guidance on MHM and safe waste management as part of the service ladders.
- Global and national platforms (e.g., conferences, networks) for advocacy on sanitation and MHM to include attention to menstrual waste management particularly through research, technology development, and intervention implementation.

The authors’ overall recommendations are also outlined in Table S6.

## 8. Conclusions

The disposal of used absorbents cannot be understood and carried out in isolation, requiring user-centered design thinking to consider socio-cultural norms regarding menstruation, product availability, quality and use, and existing sanitation systems. In institutional and community settings, the design of sanitation systems needs to be responsive to and plan for disposal of menstrual waste, incorporating it into both the sanitation and solid waste management parts of a WASH program, providing appropriate, discrete and sound solutions that minimize adverse health impacts on girls and women, sanitation workers, and broader environmental consequences. Thermal treatment is one option as a waste management and pathogen treatment approach, and with careful implementation may prove socially acceptable in many contexts, although care needs to be taken with design to ensure emissions are safe, particularly where absorbents contains chlorine or polyethene.

## Supplementary Materials

The following are available online at http://www.mdpi.com/1660-4601/15/11/2562/s1, Table S1: Summary Menstrual Hygiene Products and Absorbents used in LMIC in Africa and Asia, Table S2: Details of Literature Reviewed on Menstrual Absorbents; Table S3: Menstrual hygiene products/absorbents and the materials they are made from in LMIC; Table S4: Disposal practices reported in studies; Table S5: Overview of different incinerator technologies including type of use, advantages, disadvantaged and examples of current use. The table has been adapted from PATH (2017) [57], Table S6: Authors’ recommendations.
