# Supplementary material for: Menstrual Hygiene Management and Waste Disposal in Low and Middle Income Countries—A Review of the Literature
Source: Int J Environ Res Public Health. 2018 Nov 15;15(11):2562. doi: 10.3390/ijerph15112562 (PMC6266558; doi:10.3390/ijerph15112562)
Supplement: Supplementary file 1 [file ijerph-15-02562-s001.pdf]

Table S1. Summary Menstrual Hygiene Products and Absorbents used in LMIC in Africa and Asia.

Country	City	Age range or Mean Age (y)	Study Setting	Sample Size (*n*)	Absorbent Type<br>Type	Usage (%)	Influencing Factors	References
				Africa<br>*Northern Africa*				
Egypt	Mansoura	Menstruating	Secondary school	664	Sanitary pads (single use)	66.8	* Mass media exposure, High and middle social class, Urban residence	El-Gilany, et al, 2005 [1]
					Re-useable cloth after boiling or washing	15.9		
					Old cloth thrown away after single use	12.0		
					Other (cotton, gauze, soft tissue or nothing)	5.3		
Egypt	Zagazig city	15.813 + 0.469	Secondary School	150	Sanitary pads and Cloths	44.0		Allah and Elsabagh, 2011 [2]
					Sanitary pads only	32.0		
					Cloth only	24.0		
				Eastern Africa				
Ethiopia	Addis Ababa	16.13 ± 1.57	Secondary School	829	*Menarche Absorbent*			Abera et al., 2004 [3]
					Rag made pad	61.0		
					Commercially made pad	35.0		
					Napkin/soft paper	4.0		
					Menstrual Absorbent			
					Homemade pad	52.4		
					Commercially made	47.6		
Ethiopia	Habru, North Wollo zone	14.96 ± 1.33	School	574	Homemade cloth	55.6	*School, Residence, Living with relatives, Mother's education, Father's education, Mother's occupation, Father's occupation, Monthly expenditure	Tegegne and Sisay, 2014 [4]
					Disposal sanitary pad	35.4		
					Underwear	9.0		
Ethiopia	Tigray	10-80	Community	349	Sanitary pad (modess)	66.2	Cost	Wall et al., 2016 [5]
					Extra pants/Underwear	23.2		
					Homemade cloth	51.6		
					Sponge	0.3		
					Tissue	0.3		
Malawi	Central region	14–21	School	104	Cloth	95	Affordability, Accessibility	Pillitteri, 2011 [6]
					Sanitary pad	N.D.		

Uganda	Southern Uganda	16	Secondary school	134	Commercial disposal pads<br>Low cost pads<br>Traditional materials (cloths, leaves, newspaper, cotton wool and toilet paper)<br>Tampons	N.D<br>N.D<br>N.D<br>N.D	Cost, Absorbency, Leak-proof, Comfort, Knowledge, Accessibility	Crofts and Fisher, 2012 [7] [a]
Zambia	Mongu District	13–20	Secondary school	51	Sanitary pads<br>Cloth	N.D<br>N.D	Economic poverty, Structural and environmental poverty	Lahme et al., 2016 [8] [a]
Zimbabwe	Epworth, Harare	24	Suburban	43	Cotton wool<br>Rags/cloth/towel pieces<br>Pads<br>Tissue	53.5<br>27.9<br>16.3<br>2.3	Cost, Cleaning of products	Averbach et al., 2009 [9] [b]
<u>Western Africa</u>								
Ghana	Countrywide	10-≥20	School	229	Sanitary pads	93.5		Asimah et al., 2017 [10]
Nigeria	Onitsha	15–17	Secondary School	550	Toilet tissue paper<br>Sanitary pad<br>Clothes<br>Multiple materials<br>Tampon	41.3<br>32.7<br>14.4<br>10.7<br>0.9		Adinma and Adinma, 2008 [11]
Nigeria	Kano	14.4 ± 1.2	Secondary school	400	Sanitary pads<br>Cloth	93.5<br>6.2	*Age, Knowledge	Lawan et al., 2010 [12]
Nigeria	Sokoto	16.75 ± 1.25	Secondary school	122	Sanitary pads<br>New piece of cloth<br>Toilet paper<br>Old piece of cloth	87.0<br>8.0<br>4.0<br>1.0	*Mother's education, Religion, Mother's occupation	Oche et al., 2012 [13]
Nigeria	South-Western	15.3 ± 1.5	Secondary School	395	Sanitary pad only<br>Sanitary pad and cloth<br>Cloth only<br>Tissue paper and sanitary pad<br>Tissue paper only<br>Tampon and sanitary pad	82.3<br>10.6<br>2.3<br>2.3<br>1.8<br>0.5	*Type of school, Mother's education	Aluko et al., 2014 [12]
<u>Southern Africa</u>								
South Africa	Durban	18–45	Reproductive clinic	110	Sanitary pads<br>Panty liners	92.7<br>30.0	Financial constraints	Beksinska et al., 2015 [14] [b]

					Tampons<br>Toilet papers<br>Cloth/rag	20.0<br>17.3<br>6.4		
South Africa	Vhembe district, Limpopo province	14–17	Secondary school	273	Pieces of cloth<br>Sanitary pad<br>Hand towel<br>Newspaper	55<br>37<br>26<br>2		Ramathuba, 2015 [15]
South Africa	Durban	18–35	Communities	21	Sanitary pads	100		Scorgie et al., 2015 [16]
Asia								
South Asia								
Bangladesh	Araihazar	11–16	School	416	*Before Intervention*<br>New Cloths<br>Old Cloths/others<br>Sanitary pads<br><br>*After Intervention*<br>New Cloths<br>Sanitary pads<br>Old Cloths/others	 49.8<br>33.4<br>16.8<br><br><br>50.2<br>39.2<br>10.6		Haque, et al., 2014 [17] [b]
India	Karnataka	15–45	Urban	656	Clothes<br>Sanitary pads and clothes<br>Sanitary pads	91.46<br>4.57<br>3.96		Balamurugan and Bendigeri, 2002 [18]
India	New Delhi	26 ± 7	Urban re-settlement	254	Homemade cloth pads<br>Sanitary napkins<br>Clothes and sanitary napkins	60.2<br>28.0<br>11.0	*Economic constrains, Age, Mothers' education	Baridalyne and Reddaiah, 2004 [19]
India	Ambajogi	12–17	Urban and rural school	168	Cloth<br>Sanitary pads	N.D<br>N.D	Cost, Poverty level	Deo and Ghattargi, 2005 [20]
India	Rajasthan	15.8	School-going and out-of-school	611	Old cloth<br>Sanitary pads<br>New cloth<br>Cotton	75.6<br>19.5<br>4.1<br>0.5	*Schooling, Residential status, Caste, Occupation of father, Media exposure	Khanna et al., 2005 [21]
India	Kalamboli, Navi-mumbai	Menstruating	Secondary school	217	*Before intervention*<br>Sanitary pads and cloth<br>Sanitary pads	 43.6<br>40.6		Nemade et al., 2009 [22]

					Cloth:<br>Cotton<br>Nylon<br>Terricott<br><br>*After intervention*<br>Sanitary pads and cloth<br>Sanitary pads<br>Cloth:<br>Cotton<br>Terricott<br>Nylon	15.7<br>70.1<br>7.7<br>22.2<br><br><br>N.D<br>N.D<br>N.D<br>94.0<br>4.3<br>2.7		
India	South India	18.6 ± 1.7	Educational institutions	350	Sanitary napkins<br>Cloth material<br>Cotton disposable<br>Others	68.9<br>19.1<br>7.4<br>3.7	*Age, Socioeconomic status	Omidvar and Begum, 2010 [23]
India	Mumbai	10–19	Urban slum	241	Sanitary pads<br>Old cloth pieces<br>Others<br>New cloth pieces	43.2<br>30.7<br>15.4<br>10.8	*Knowledge, Education	Bobhate et al., 2011[24]
India	Ranchi	11–20	School	117	Cloth<br>Sanitary pads	N.D<br>N.D	Residential status	Kumar and Srivastava, 2011 [25]
India	Saoner, Nagpur district	13.82 ± 0.832	School	Urban= 241	Sanitary pad<br>Old cloth<br>New cloth	60.6<br>35.7<br>3.7	Knowledge, Cost, availability, Shyness, Others (mother's restriction, disposal problems)	Thakre et al., 2011 [26]
India	Amritsar, Punjab	10–15	School	300	Sanitary pads<br>New cloth pieces and pads<br>Any cloth or rag or cotton	69.0<br>21.0<br>10.0		Kamaljilt et al., 2012 [27]
India	Thiruvananthapuram, Kerala	15–19	Secondary school	3,443	Sanitary pad<br>Sanitary pad and cloth<br>Cloth	45.5<br>38.2<br>16.3		Nair et al., 2012 [28]
India	Pune	10–19	Secondary school	622	Cloth (new+old)<br>Sanitary pads	51.9<br>48.0		Bodat et al., 2013 [29]
India	Mumbai	15–24	Community	96	Sanitary pads/ combination with cloth<br>Cloth	74.5<br>25.0		Thakur et al., 2014 [30]

India	Countrywide	15–49	Census data	643,944	Cloth<br>Sanitary napkins<br>Locally prepared napkins<br>Nothing<br>Other	90.1<br>11.2<br>3.9<br>1.3<br>0.2	*Higher secondary education, Married after age 18, Residing in urban area, Christian religion, Wealth index, Access to toilet, Mass media exposure	Anand et al., 2015 [31]
India	Kolkata, West Bengal	14.2 ± 1.5	Urban, rural	715	Sanitary pads<br>Clothes	83.1<br>16.9	*Age, Education, Mother's education, Father's education, Residential status, Household income	Mishra et al., 2017 [32]
Nepal	Dhading, Morang, Lalitpur and Kathmandu	12–20	Urban and rural Secondary school	174	Sanitary pad<br>New piece of cloth<br>Old piece of cloth	50.0<br>36.0<br>14.0	Affordability, Availability, Difficulty in disposal, Knowledge	Wateraid, 2009 [33]
Pakistan	Karachi	12–19	Government Schools	425	Pieces of old clothes<br>Sanitary pads<br>Pieces of new clothes<br>Cotton wool<br>Woolen cloth<br>Shalwar/nothing	50.0<br>33.5<br>10.6<br>4.4<br>1.4	*Status of schooling	Ali and Rizvi, 2010 [34]
			Private Schools	465	Pieces of old clothes<br>Pieces of new clothes<br>Sanitary pads<br>Cotton wool<br>Woollen cloth<br>Shalwar/nothing	62.6<br>16.8<br>16.4<br>3.6<br>0.6		
			Communities	433	Pieces of old clothes<br>Sanitary pads<br>Pieces of new clothes<br>Cotton wool<br>Woollen cloth<br>Shalwar/nothing	70.4<br>13.0<br>8.0<br>6.5<br>1.1<br>1.1		
				Southeast Asia				
Philippines	Masbate Province and Metro Manila	11–18	Urban and rural schools	68	Sanitary pads<br>Cloth or old clothing	N.D<br>N.D	Accessibility to preferred materials, Knowledge and WASH facilities	Haver et al., 2013 [35] [a]
				Western Asia/Middle East				
Iran	Tehran	11–15	Middle school	1823	Sanitary pads or Cotton	27.4	* Training	Djalalinia et al., 2012 [36] [b]

N.D.: No Data; N/A: Not Applicable; *: statistically significant association between factors and hygienic practices (include using sanitary pads)/sanitary materials use; a: Qualitative studies/reports; b: Intervention studies.

Table S2. Details of Literature Reviewed on Menstrual Absorbents

**First Author / Org Name**	**Year**	**Title**	**Study Type/ Research Approach**	**Abstract**
N. Baridalyne and V. P. Reddaiah	2004	Menstruation: Knowledge, beliefs and practices of women in the reproductive age group residing in an urban resettlement colony of Delhi	Cross-sectional, Descriptive; Mixed method	In a community-based cross-sectional study, knowledge, beliefs and practices on menstruation were studied among the reproductive age group women residing in an urban resettlement colony of Delhi. Though menstruation is a physiological process, 86 percent of women were not psychologically prepared for it. As an absorbent, home-made pads made of unwashed clothes were used by 60 percent of women and three-fourths of them were unaware of the problems caused as a result of using dirty clothes. The quality of absorbent material used was significantly related to age and maternal education of the subjects. 79 percent of the subjects admitted that excessive bleeding and severe abdominal pain were abnormal. For those women having problems (28%) in the last menstrual period, 44 percent reported excessive bleeding while 17 percent had severe abdominal pain. While 38 percent did not seek any remedial measures, 45 percent consulted a doctor and 15 percent took self-medication. The above findings highlight the need for health education among women so as to increase awareness and correct knowledge regarding various aspects of menstruation and menstrual hygiene.
O. O. Aluko, O. M. Oluya, O. A. Olaleye, A. A. Olajuyin, T. F. Olabintan and O. I. Oloruntoba-Oju	2014	Knowledge and menstrual hygiene practices among adolescents in senior secondary schools in Ile Ife, south-western Nigeria	Cross-sectional, Descriptive, Mixed method	A culture of silence surrounds menstruation while inadequate facilities predispose adolescents to psycho-social trauma and cyclic absenteeism from schools. This study assessed the knowledge and menstrual hygiene management (MHM) practices among in-school adolescents in an urban city in Nigeria. The descriptive, cross-sectional study identified 400 respondents through a multistage technique and collected data through validated questionnaire and observational checklist. The mean age and age-at-menarche of respondents were 15.3 +/- 1.5 and 12.8 years, respectively. Most respondents (70%) were aged 10-15 years, had good knowledge of MHM (296, 74%) and knew about menstruation before menarche (85.4%). MHM knowledge was significantly associated with: mother's education (p = 0.029); absorbents changing frequency (p = 0.003); and age-at-menarche (p = 001). The number of absorbents used daily was 2.5 +/- 0.7; 90% of adolescents changed absorbents at least twice daily while 24.2% had previously changed it in school. Moreover, 14.4% of respondents abstained from school during menstruation and there was a significant association between school type and menstrual absorbents used (p = 0.0001), mothers' education (p = 0.0001) and disposal of used absorbents (p = 0.004). Spent absorbents were mostly disposed of in pit latrines (35.1%) and by burning (32.6%). A wide disparity remains between good MHM knowledge and poor practices. Therefore, gender-friendly facilities should be provided in schools to ensure retention of girls and end psycho-social trauma experienced during menstruation.

T. Crofts and J. Fisher	2012	Menstrual hygiene in Ugandan schools: an investigation of low-cost sanitary pads	Exploratory, Qualitative	Menstrual hygiene management (MHM) is a largely overlooked issue in the water, sanitation and hygiene (WASH) sector. Every day, millions of menstruating girls and women in low-income countries struggle to find clean water for washing, private places for changing and adequate blood absorbing materials. This study aims to explore the difficulties experienced by schoolgirls in Uganda in managing menstrual hygiene and investigates the extent to which low-cost sanitary pads are part of the solution. Low-cost sanitary pads, either re-usable or disposable, are a timely, simple and innovative means of improving menstrual hygiene and of addressing a broader set of problems related to MHM in schools. Other factors highlighted are: pain relief, education, safe water provision, clean and private latrines, hygienic and secure bathing facilities, use of soap, sealed waste disposal points, private drying places, anal cleansing materials and effective facility operation and management strategies.
S. Bharadwaj and A. Patkar	2004	Menstrual hygiene and management in developing countries: Taking stock	Exploratory	Across the developing world, the lack of appropriate and adequate sanitation facilities prevents girls from attending school, particularly when they are menstruating. Of the 113 million children currently not enrolled in school worldwide, 60% are girls. The literature on gender mainstreaming in the water & sanitation sector is silent on menstrual management, adequacy of water for washing and bathing, availability of hygienic materials and solid waste management of disposables. Initiatives in this area are restricted to very small pilots, with poor follow-up and poor dissemination of results. Although poor sanitation is correlated with absenteeism and drop-out of girls in developing countries, efforts in school sanitation to address this issue have ignored menstrual management in latrine design and construction. Minimal effort has gone into production and social marketing of low-cost napkins, reusable materials, research into bio-degradables, etc. Wider aspects of the issue, such as privacy, water availability and awareness-raising amongst boys and men remain largely unexplored by development initiatives. This paper collates the findings from a serious effort to take stock of the current thinking, practices, barriers, investments and action linked to menstrual hygiene management. It is based on wide electronic consultation and secondary desk review with key stakeholders in the area of health, hygiene, water and sanitation and women's rights and incorporates the knowledge and experience from more than a decade of first-hand experience in water, sanitation and reproductive health in developing countries. The overall objective of this paper was to compile a brief overview of initiatives in menstrual management as a precursor to action.
R. Wilmouth, N. Muller, C. Truyens, L. Hoppenjans, M. Sommer, S. Adelman and V. Hoffmann	2013	Interactions between menstrual hygiene management and sanitation systems: Landscape analysis of menstrual hygiene products and a waste-loading model	Modelling	To ensure successful integration of modern menstrual hygiene products (MHPs) with sanitation systems, it is important to understand what is currently done with menstrual waste in urban and peri-urban settings. This is particularly true in light of global trends towards urbanization and the increasing availability of disposable sanitary pads in these settings. The overall Menstrual Management & Sanitation Systems Project is led by the University of Maryland. To support the larger project and address the lack of existing research on women's menstrual hygiene management (MHM), PATH conducted two case studies in South Africa and India from March–December 2012.

V. Goyal	2016	Scope and Opportunities for Menstrual Health and Hygiene Products in India	Exploratory	Menstrual hygiene continues to be amongst the most challenging development issues today. Not only do deep-rooted taboos, myths and misinformation create the illusion that menstruation is inherently shameful, gross and weird, but in countries like India, women and girls often lack access to hygienic sanitary materials and basic facilities Sanitary Pads necessary for good menstrual hygiene management (MHM). "Sanitary Protection: Every Woman's Health Right "a study by AC Nielsen reveals only 12 % of India's 355 million women use sanitary napkins. Over 88% of women resort to shocking alternatives like un-sanitized cloth, ashes and husk sand. Incidents of Reproductive Tract Infection (RTI) are 70% more common among these women1. The biggest barrier to using a Sanitary Napkin (SN) is affordability. Around 70% of women in India say their family can't afford to buy them, 88% of women use old fabric, rags or sand to manage their flow. This tells the immense entrepreneurial opportunities in the segment. A recent study anticipate the industry to grow to INR 45.9 billion by 2017.the only need to work in this direction is the reduction in cost of production.
D. Ssewanyana and B. K. Y. Bitanihirwe	2017	Menstrual hygiene management among adolescent girls in sub-Saharan Africa	Review, Exploratory	Menstruation is a delicate physiological process through which a shedding of uterine lining occurs each month in females of reproductive age. Often considered a taboo subject, menstruation is seldom openly discussed in developing parts of the world. This article explores menstrual hygiene management (MHM) in sub-Saharan Africa and emphasizes the urgent and neglected need for feasible solutions, especially among adolescent girls. Optimizing menstrual hygiene interventions will require an integration of both knowledge and skill training gained through education on MHM alongside an improvement of access to girl-friendly water, sanitation and hygiene facilities in addition to access to low-cost hygienic sanitary products. To facilitate the identification and implementation of feasible and cultural relevant programs we recommend the utilization of public health intervention research.
L. L. Wall, S. Belay, A. Bayray, S. Salih and M. Gabrehiwot	2016	A community-based study of menstrual beliefs in Tigray, Ethiopia	Cross-sectional, Mixed	OBJECTIVE: To investigate knowledge and beliefs about menstruation in the Tigray Region of Ethiopia. METHODS: Between May 5 and May 25, 2015, a cross-sectional survey using semi-structured questionnaires was undertaken in 10 subdistricts (5 urban, 5 rural) in the Tigray Region of northern Ethiopia by trained data collectors (native speakers of the local languages). Individuals in randomly selected households who were aged 10years or older and who were willing to participate were asked various questions regarding the nature and management of menstruation. Interviews were recorded, and handwritten field notes were taken during the interview process. Data were compiled, transcribed, translated into English, categorized, and analyzed thematically. RESULTS: Overall, 428 household members (349 female, 79 male) were interviewed. Reproductive anatomy and biology of menstrual regulation were poorly understood by the respondents. The belief that menstruating girls should not attend school was voiced by 17 (21.5%) male and 37 (10.6%) female respondents. Satisfactory management of menstrual hygiene was acknowledged to be a problem, and many respondents complained about the high cost of commercially produced, disposable menstrual pads. CONCLUSION: Improved education on menstruation and better access to low-cost, reusable menstrual hygiene supplies would be worthwhile in the Tigray Region of Ethiopia.

A. M. Lahme, R. Stern and D. Cooper	2016	Factors impacting on menstrual hygiene and their implications for health promotion	Exploratory	BACKGROUND: In the lives of women, puberty is marked by the onset of menarche. From this stage onwards until menopause, reproductive health and menstrual hygiene are important aspects of women's lives. In Zambia's Western Province, the natural process of menstruation is a taboo and dealt with secretly. Information and knowledge about menstruation and menstrual hygiene among adolescent girls is inadequate. This paper explores the factors influencing the understanding, experiences and practices of menstrual hygiene among adolescent girls in Mongu District, Western Province of Zambia. METHODS: An explorative study design was used by means of six focus group discussions conducted with 51 respondents, aged 13-20 years, from three secondary schools. Their age at menarche was 11-15. For data analysis thematic content analysis was used. RESULTS: The paper shows that the girls suffer from poor menstrual hygiene, originating from lack of knowledge, culture and tradition, and socio-economic and environmental constraints, leading to inconveniences, humiliation and stress. This leads to reduced school attendance and poor academic performance, or even drop outs, and ultimately infringes upon the girls' human rights. CONCLUSION: To address these shortcomings, a 'super setting approach' is recommended, in which a Health Promoting School could improve the girls' individual and group needs, and a community setting which would address the broader socio-economic, cultural and environmental conditions. This would enable creating a supportive environment for the girls to manage their periods. To successfully utilize the approach, all stakeholders (parents, teachers, children, governments and communities) should cooperate to generate context-specific solutions for creating safe menstrual care, and better and dignified conditions for adolescent girls. Therefore, this calls for comprehensive, strident advocacy for policy changes at national level, and mediation and involvement at community level.
H. Thakur, A. Aronsson, S. Bansode, C. Stalsby Lundborg, S. Dalvie and E. Faxelid	2014	Knowledge, Practices, and Restrictions Related to Menstruation among Young Women from Low Socioeconomic Community in Mumbai, India	Mixed method	The main objective was to assess knowledge, practices, and restrictions faced by young women regarding their menstrual hygiene. The views of adult women having young daughters were also included and both views were compared. In addition, the factors influencing the menstrual hygiene practices were also studied. The study was carried out during 2008 in Mumbai, India. The mixed methods approach was followed for the data collection. Both qualitative and quantitative methods were used to collect the data. For quantitative survey, totally 192 respondents (96 adult and 96 younger women) were selected. While young women were asked about questions related to their menstruation, adult women were asked questions to find out how much they know about menstrual history of their daughters. The qualitative data helped to supplement the findings from the quantitative survey and to study the factors affecting menstrual practices in young women. The mean age at menarche reported was 13.4 years and 30-40% of young girls did not receive any information about menstruation before menarche. It is thus seen that very few young girls between the age group 15 and 24 years did receive any information before the onset of menstruation. Among those who received some information, it was not adequate enough. The source of information was also not authentic. Both young and adult women agreed on this. Due to the inadequate knowledge, there were certain unhygienic practices followed by the young girls resulting in poor menstrual hygiene. It also leads to many unnecessary restrictions on young girls and they faced many health problems and complaints, which were either ignored or managed inappropriately. The role of health sector was almost negligible from giving information to the management of health problems of these young girls. This paper reemphasizes the important, urgent, and neglected need of providing correct knowledge to the community including adolescent girls.

T. S. Ali and S. N. Rizvi	2010	Menstrual knowledge and practices of female adolescents in urban Karachi, Pakistan	Cross-sectional	Menstruation is a normal physiological process that is managed differently according to various social and cultural understandings. Therefore, this cross-sectional study was conducted to explore the menstrual practices among 1275 female adolescents of urban Karachi, Pakistan from April to October 2006 by using interviews. Data was entered and analyzed in Epi Info Version 9 and SPSS Version 10. Descriptive findings showed that 50% of the girls lacked an understanding of the origin of menstrual blood and those with a prior knowledge of menarche had gained it primarily through conversations with their mothers. Many reported having fear at the first experience of bleeding. Nearly 50% of the participants reported that they did not take baths during menstruation. In univariate analysis, factors of using unhygienic material, using washcloths, and not drying under sun were found to be significant in the Chi square test among those going and not going to schools. This study concludes that there are unhygienic practices and misconceptions among girls requiring action by health care professionals.
S. E. Haque, M. Rahman, K. Itsuko, M. Mutahara and K. Sakisaka	2014	The effect of a school-based educational intervention on menstrual health: an intervention study among adolescent girls in Bangladesh	Intervention	OBJECTIVES: To assess the impact of a school-based menstrual education programme on: (1) menstrual knowledge, beliefs and practices, (2) menstrual disorders experienced, and (3) restrictions on menstruating adolescents. DESIGN: Intervention study. SETTING: Araihazar area, Bangladesh. PARTICIPANTS: 416 adolescent female students aged 11-16 years, in grade 6-8, and living with their parents. INTERVENTIONS: A school-based health education study conducted from April 2012 to April 2013. PRIMARY AND SECONDARY OUTCOME MEASURES: We randomly selected 3 of 26 high schools in the study area. We delivered 6 months of educational intervention by trained (by an obstetrician and gynaecologist) research assistants (RAs) on menstrual hygiene among school girls. RAs read the questionnaire and participants answered. The changes in knowledge, beliefs and practices regarding menstruation, menstrual disorders experienced, and the restrictions and behaviours practiced by menstruating adolescents were compared between the baseline and the follow-up assessments. RESULTS: After health education, participants reported a significant improvement ($p<0.001$) in 'high knowledge and beliefs' scores compared to baseline (51% vs 82.4%). Significant improvement was also observed in overall good menstrual practices (28.8% vs 88.9%), including improvements in using sanitary pads (22.4% change after the intervention), frequency of changing pads/cloths per day (68.8%), drying the used absorbent (77.6%), methods of disposing of the used absorbent (25.5%), and cleaning of genitalia (19.2%). During the follow-up, the participants reported significant improvements in the regularity of their menstrual cycle (94.5% vs 99.5%) and fewer complications during menstruation (78.6% vs 59.6%). CONCLUSIONS: The programme produced significant changes in the knowledge, beliefs and practices of menstrual hygiene, complications from lack of hygiene, and the behaviour and restrictions of the menstruating adolescents. These results demonstrate the feasibility of implementing a health education programme for adolescents on menstrual hygiene in secondary schools serving rural Bangladesh.

A. H. El-Gilany, K. Badawi and S. El-Fedawy	2005	Menstrual hygiene among adolescent schoolgirls in Mansoura, Egypt	Cross-sectional	Learning about menstrual hygiene is a vital aspect of health education for adolescent girls. This study among 664 schoolgirls aged 14-18 in Mansoura, Egypt, asked about type of sanitary protection used, frequency of changing pads or cloths, means of disposal and bathing during menstruation. Girls were selected by cluster sampling technique in public secondary schools in urban and rural areas. Data were collected through an anonymous, self-administered, open-ended questionnaire during class time. The significant predictors of use of sanitary pads were availability of mass media at home, high and middle social class and urban residence. Use of sanitary pads may be increasing, but not among girls from rural and poor families, and other aspects of personal hygiene were generally found to be poor, such as not changing pads regularly or at night, and not bathing during menstruation. Lack of privacy was an important problem. Mass media were the main source of information about menstrual hygiene, followed by mothers, but a large majority of girls said they needed more information. Instruction in menstrual hygiene should be linked to an expanded programme of health education in schools. A supportive environment for menstrual hygiene has to be provided both at home and in school and sanitary pads made more affordable.
S. K. Mishra, D. Dasgupta and S. Ray	2016	A study on the relationship of sociocultural characteristics, menstrual hygiene practices and gynaecological problems among adolescent girls in Eastern India	Cross-sectional	OBJECTIVE: The present study aims to understand the relationship of socioeconomic characteristics, menstrual hygiene practices and gynaecological problems among adolescent girls residing in rural and urban areas in the state of West Bengal, India. METHODS: The study was based on a sample of 715 adolescent girls from rural (325) and urban (390) areas of West Bengal, a state in Eastern India. These girls belong to the Bengali-speaking Hindu community. Data on socioeconomic characteristics, menstrual hygiene practices (such as type of absorbents used and mode of cleaning of genitals during days of menstrual discharge) and gynaecological problems were collected using pretested questionnaires. RESULTS: Rural and urban girls differ ($p<0.01$) for age at menarche, menstrual hygiene practices and prevalence of gynaecological problems. Urban girls have better menstrual hygiene practices (beta=0.343, $p<0.01$) than rural girls. A similar trend is noted for gynaecological problems (beta=0.080, $p<0.01$) among the study participants. Apart from socioeconomic characteristics, menstrual hygiene (beta=-0.121, $p<0.01$) remains a significant predictor of gynaecological problems. The results of path analysis also indicate that girls of higher socioeconomic status have better menstrual hygiene practices which subsequently reduce the prevalence of gynaecological problems among them. CONCLUSION: A concerted effort from parents, educational institutions and existing healthcare institutions along with media may ensure safe and secure reproductive health prospects for adolescents in the region.

D. Shanbhag, R. Shilpa, N. D'Souza, P. Josephine, J. Singh and B. R. Goud	2012	Perceptions regarding menstruation and practices during menstrual cycles among high school going adolescent girls in resource limited settings around Bangalore city, Karnataka, India	Cross-sectional	Introduction: Hygiene-related practices of adolescents during menstruation are of importance, as it has a health impact in terms of increased vulnerability to reproductive tract infections (RTI). Therefore, increased knowledge about menstruation right from childhood may escalate safe practices and may help in mitigating the suffering of women. Objectives: To assess the perceptions and practices regarding menstrual hygiene among selected high school girls in a resource limited settings in area around Bangalore city. Methodology: This was a cross sectional study done in four selected Government High Schools in rural areas around Bangalore City. A pre-designed, pre-tested and structured questionnaire was administered. Results: A total of 506 girls were interviewed. The average age was 14.08 with Standard deviation of 1.06 and range between 12-16yrs. 99.6% of the students had heard of menstruation and 57.9% had acquired this even knowledge before attaining menarche. 73.7% knew that menstruation was a normal phenomenon but only 28.7% had knowledge regarding menstruation. 48.1% did not know that menstruation was related to pregnancy. Only 44.1% used sanitary pad during the menstrual cycles. Among those who used cloth, only 31.3% used soap and water to clean them. 56.8% used soap and water to clean their genital organs and 88.8% of the girls took bath daily during menstruation.
K. Kamaljit, A. Balwinder, K. S. Gurmeet and N. S. Neki	2012	Social beliefs and practices associated with menstrual hygiene among adolescent girls of Amritsar, Punjab, India	Exploratory, Descriptive	Menstrual practices are still shrouded by taboos and socio-cultural restrictions. Thus adolescent girls remain ignorant of the scientific facts and hygiene practices which sometimes result into adverse health consequences. This study was undertaken with the objective of eliciting the beliefs, perception and source of information regarding menstruation among the adolescent girl students and to find out the status of menstrual hygiene among adolescent girls. An exploratory and descriptive study has been conducted among 10th clas adolescent girl students of senior secondary schools in Amritsar City, Punjab. A pre-designed and pretested interview schedule has been during the interview. Data has been analyzed statistically by chi square test. Out of 300 respondents 184(61.3percent) adolescent girls are having awareness about menstruation prior to initiation of menarche. Mother was the first informant regarding menstruation in case of 160 (53.3 per cent) girls. 221 (73.7 per cent) girls believed it a normal physiological process. 207 (69.0 per cent) girls knew regarding use of sanitary ad during the menstruation. Regarding menstrual hygiene practices, 68.7 per cent girls used sanitary pads and 30 (10.0 per cent) respondents practicing any cloth or rag/ cotton during menstruation. For the purpose of cleaning external practiced by the family, 294 (98.0 per cent) girls practiced different types of family restrictions during menstruation.

S. B. Thakre, S. S. Thakre, M. Reddy, N. Rathi, K. Pathak and S. Ughade	2011	Menstrual hygiene: Knowledge and practice among adolescent school girls of Saoner, Nagpur District	Cross-sectional	Background: Menstruation is generally considered as unclean in the Indian society. Isolation of the menstruating girls and restrictions being imposed on them in the family, have reinforced a negative attitude towards this phenomenon. There is a substantial lacuna in the knowledge about menstruation among adolescent girls. Good hygienic practices such as the use of sanitary pads and adequate washing of the genital area are essential during menstruation. Menstrual hygiene and management will directly contribute to the Millennium Development Goal (MDG)-2 on universal education and MDG -3 on gender equality and women empowerment. Aim and Objectives: To assess the knowledge and the practices of menstrual hygiene among rural and urban school going adolescent girls. Materials and Methods: A community based, cross sectional study was conducted in January- March, 2011 on 387 school going girls. The present study was undertaken among adolescent school going girls in the field practice area of the Rural Health Unit and Training Centre, Saoner, in the Nagpur district. Three hundred and eighty seven girls of the 8th and 9th standards were purposively selected for the study. A pre-designed, pretested and structured questionnaire was used in the study. The data collection technique was a personal interview of the study subjects. Results: Only 36.95% of the girls were aware of menstruation before menarche. The major source of information about menstruation for them was found to be their mothers. More than three fourth of the girls in the study were not aware of the cause and the source of the bleeding. A majority of them had knowledge about the use of sanitary pads. The mean age of menarche in the study subjects was 12.85 ± 0.867 years; sanitary pads were used by 49.35% of the selected girls. The practice of the use of old clothes was reported in 45.74% of the subjects. Satisfactory cleaning of the external genitalia was practised by 33.85% of the girls. Three fourth of the study girls practised various restrictions during menstruation. Some menstrual hygiene indices have shown a significant difference in the rural and urban girls. Conclusion: A variety of factors are known to affect menstrual behaviours, the most influential being economic status and residential status (urban and rural). Awareness regarding the need for information about healthy menstrual practices is very important. It is essential to design a mechanism to address and for the access of healthy menstrual knowledge.
A. M. van Eijk, M. Sivakami, M. B. Thakkar, A. Bauman, K. F. Laserson, S. Coates and P. A. Phillips-Howard	2016	Menstrual hygiene management among adolescent girls in India: a systematic review and meta-analysis	Systematic review with meta-analysis	OBJECTIVES: To assess the status of menstrual hygiene management (MHM) among adolescent girls in India to determine unmet needs. DESIGN: Systematic review and meta-analysis. We searched PubMed, The Global Health Database, Google Scholar and references for studies published from 2000 to September 2015 on girls' MHM. SETTING: India. PARTICIPANTS: Adolescent girls. OUTCOME MEASURES: Information on menarche awareness, type of absorbent used, disposal, hygiene, restrictions and school absenteeism was extracted from eligible materials; a quality score was applied. Meta-analysis was used to estimate pooled prevalence (PP), and meta-regression to examine the effect of setting, region and time. RESULTS: Data from 138 studies involving 193 subpopulations and 97,070 girls were extracted. In 88 studies, half of the girls reported being informed prior to menarche (PP 48%, 95% CI 43% to 53%, I(2) 98.6%). Commercial pad use was more common among urban (PP 67%, 57% to 76%, I(2) 99.3%, n=38) than rural girls (PP 32%, 25% to 38%, I(2) 98.6%, n=56, p<0.0001), with use increasing over time (p<0.0001). Inappropriate disposal was common (PP 23%, 16% to 31%, I(2) 99.0%, n=34). Menstruating girls experienced many restrictions, especially for religious activities (PP 0.77, 0.71 to 0.83, I(2) 99.1%, n=67). A quarter (PP 24%, 19% to 30%, I(2) 98.5%, n=64) reported missing school during periods. A lower prevalence of absenteeism was associated with higher commercial pad use in univariate (p=0.023) but not in multivariate

				analysis when adjusted for region (p=0.232, n=53). Approximately a third of girls changed their absorbents in school facilities (PP 37%, 29% to 46%, I(2) 97.8%, n=17). Half of the girls' homes had a toilet (PP 51%, 36% to 67%, I(2) 99.4%, n=21). The quality of studies imposed limitations on analyses and the interpretation of results (mean score 3 on a scale of 0-7). CONCLUSIONS: Strengthening of MHM programmes in India is needed. Education on awareness, access to hygienic absorbents and disposal of MHM items need to be addressed. TRIAL REGISTRATION NUMBER: CRD42015019197.
A. S. Kuhlmann, K. Henry and L. L. Wall	2017	Menstrual Hygiene Management in Resource-Poor Countries	Review	Importance: Adequate management of menstrual hygiene is taken for granted in affluent countries; however, inadequate menstrual hygiene is a major problem for girls and women in resource-poor countries, which adversely affects the health and development of adolescent girls. Objective: The aim of this article is to review the current evidence concerning menstrual hygiene management in these settings. Evidence Acquisition: A PubMed search using MeSH terms was conducted in English, supplemented by hand searching for additional references. Retrieved articles were reviewed, synthesized, and summarized. Results: Most research to date has described menstrual hygiene knowledge, attitudes, and practices, mainly in sub-Saharan Africa and South Asia. Many school-based studies indicate poorer menstrual hygiene among girls in rural areas and those attending public schools. The few studies that have tried to improve or change menstrual hygiene practices provide moderate to strong evidence that targeted interventions do improve menstrual hygiene knowledge and awareness. Conclusion and Relevance: Challenges to improving menstrual hygiene management include lack of support from teachers (who are frequently male); teasing by peers when accidental menstrual soiling of clothes occurs; poor familial support; lack of cultural acceptance of alternative menstrual products; limited economic resources to purchase supplies; inadequate water and sanitation facilities at school; menstrual cramps, pain, and discomfort; and lengthy travel to and from school, which increases the likelihood of leaks/stains. Areas for future research include the relationship between menarche and school dropout, the relationship between menstrual hygiene management and other health outcomes, and how to increase awareness of menstrual hygiene management among household decision makers including husbands/fathers and in-laws.

C. Sumpter and B. Torondel	2013	A systematic review of the health and social effects of menstrual hygiene management	Systematic review	BACKGROUND: Differing approaches to menstrual hygiene management (MHM) have been associated with a wide range of health and psycho-social outcomes in lower income settings. This paper systematically collates, summarizes and critically appraises the available evidence. METHODS: Following the PRISMA guidelines a structured search strategy was used to identify articles investigating the effects of MHM on health and psycho-social outcomes. The search was conducted in May 2012 and had no date limit. Data was extracted and quality of methodology was independently assessed by two researchers. Where no measure of effect was provided, but sufficient data were available to calculate one, this was undertaken. Meta-analysis was conducted where sufficient data were available. RESULTS: 14 articles were identified which looked at health outcomes, primarily reproductive tract infections (RTI). 11 articles were identified investigating associations between MHM, social restrictions and school attendance. MHM was found to be associated with RTI in 7 papers. Methodologies however varied greatly and overall quality was low. Meta-analysis of a subset of studies found no association between confirmed bacterial vaginosis and MHM (OR: 1.07, 95% CI: 0.52-2.24). No other substantial associations with health outcomes were found. Although there was good evidence that educational interventions can improve MHM practices and reduce social restrictions there was no quantitative evidence that improvements in management methods reduce school absenteeism. CONCLUSION: The management of menstruation presents significant challenges for women in lower income settings; the effect of poor MHM however remains unclear. It is plausible that MHM can affect the reproductive tract but the specific infections, the strength of effect, and the route of transmission, remain unclear. There is a gap in the evidence for high quality randomised intervention studies which combine hardware and software interventions, in particular for better understanding the nuanced effect improving MHM may have on girls' attendance at school.
V. Chandra-Mouli and S. V. Patel	2017	Mapping the knowledge and understanding of menarche, menstrual hygiene and menstrual health among adolescent girls in low- and middle-income countries	Review	Background: Menstruation is a natural physiological process that requires proper management. Unlike other normal bodily processes, menstruation is linked with religious and cultural meanings that can affect the perceptions of young girls as well as the ways in which the adults in the communities around them respond to their needs. Objectives: This review aims to answer the following questions: (1) how knowledgeable are adolescent girls in low-and middle-income countries about menstruation and how prepared are they for reaching menarche, (2) who are their sources of information regarding menstruation, (3) how well do the adults around them respond to their information needs, (4) what negative health and social effects do adolescents experience as a result of menstruation, and (5) how do adolescents respond when they experience these negative effects and what practices do they develop as a result? Methods: Using a structured search strategy, articles that investigate young girls' preparedness for menarche, knowledge of menstruation and practices surrounding menstrual hygiene in LMIC were identified. A total of 81 studies published in peer-reviewed journals between the years 2000 and 2015 that describe the experiences of adolescent girls from 25 different countries were included. Results: Adolescent girls in LMIC are often uninformed and unprepared for menarche. Information is primarily obtained from mothers and other female family members who are not necessarily well equipped to fill gaps in girls' knowledge. Exclusion and shame lead to misconceptions and unhygienic practices during menstruation. Rather than seek medical consultation, girls tend to miss school, self-medicate and refrain from social interaction. Also problematic is that relatives and teachers are often not prepared to respond to the needs of girls. Conclusion: LMIC must recognize that lack of preparation, knowledge and poor practices surrounding menstruation are key

				impediments not only to girls' education, but also to self-confidence and personal development. In addition to investment in private latrines with clean water for girls in both schools and communities, countries must consider how to improve the provision of knowledge and understanding and how to better respond to the needs of adolescent girls.
M. Sommer, M. Kjellen and C. Pensulo	2013	Girls' and women's unmet needs for menstrual hygiene management (MHM): the interactions between MHM and sanitation systems in low-income countries	Review	While the sanitation sector is gaining increased recognition in policy and research, its inherent inter-linkage with menstrual hygiene management remains an under-researched subject. This review explores knowledge about menstrual beliefs and behaviors, and how women and girls currently handle their monthly menses in relation to existing sanitation systems in low-income countries. It further explores how used menstrual materials are disposed of, and the consequences of different disposal practices for the functioning of sanitation systems. Conclusions point towards the inadequacy of research in the area of menstrual management. The lack of privacy and space for changing, cleaning, drying or discarding materials, as well as insufficient availability of water for personal hygiene stand out as important areas where sanitation systems often fail to cater to the needs of menstruating girls and women. Information on proper disposal of menstrual materials as well as the actual provision of disposal facilities are important for improving menstrual management and ensuring that absorption materials do not impair the functioning of sanitation systems. Training of sanitation system designers and planners with regard to menstrual management could lead to sanitation systems becoming more inclusive of the full needs of all people.
E. Tilley, S. Bieri and P. Kohler	2013	Sanitation in developing countries: a review through a gender lens	Review	Sanitation has evolved from a purely technical discipline to one that includes social, environmental, economic and, increasingly, gender considerations. However, blurry notions of gender are frequently offered in the sanitation literature. Although it has been recognized that gender-responsive sanitation does not mean 'toilets for women', substantial alternatives are rarely debated. We structure our review of sanitation in developing countries along three lines: we start by fine-tuning the concept of gender both from the academic and the practitioner's perspective, analyse relevant developments in gender-specific policies and programming, and finally review the most appropriate toilet room and menstrual hygiene technologies. We argue that strategies to make technologies gender-responsive need to be based upon a thorough analysis of the social arrangements of the intimate, and how these are negotiated and institutionalized in a specific context. A lack of robust gender-segregated data on sanitation policies and technologies, along with reductionist framings of gender are to blame for limited progress in verifying the

				need for, and impact of, gender-responsive sanitation. Technology and policy development and implementation would benefit from gender-considerate interpretations of shame, dignity, safety and status. Further progress could be achieved by improving the translation process between different academic framings of the sanitation crisis.
Sommer M	2014	A comparison of the menstruation and education experiences of girls in Tanzania, Ghana, Cambodia and Ethiopia	Comparative-Case	The barriers to menstrual hygiene management faced by adolescent schoolgirls in low-income countries are gaining interest at practice and policy levels. The challenges include inadequate water, sanitation and disposal facilities for the management of menses with privacy and dignity, and insufficient guidance to help girls feel confident in attending school during menses. The studies described here aimed to examine how menarche impacts the lives of schoolgirls in three low-income countries (Ghana, Cambodia and Ethiopia). The focus included girls' school participation; their relationship with parents, teachers and peers; their evolving sanitation and hygiene needs; their understanding of cultural issues and taboos around menses; and what education, if any, they received prior to and during puberty. This comparative analysis was aimed at identifying similarities between the three countries that would enable the adaptation to each context of a training book on menstruation issues for girls, which was developed from a previous study conducted in Tanzania. In all three countries, participatory activities were utilised with girls and results were analysed using grounded theory. Findings included: similarities regarding the importance of culture in perpetuating negative attitudes towards menstruation, limited provision of health information and insufficient facilities within schools. Differences were revealed regarding menstrual myths, parent–child dynamics, sources of guidance and student–teacher relations. There is a critical knowledge gap around menstruation and girls' education in these contexts that must be addressed to ensure that girls experience a positive menarche and can manage menstrual hygiene. © 2014 British Association for International and Comparative Education.
Deo	2005	Perceptions and practices regarding menstruation: A comparative study in urban and rural adolescent girls	Cross-sectional, Comparative-Case	Adolescence in girls is a turbulent period, which includes stressful events like menarche, considered as a landmark of female puberty1 . One might expect that menarche will be positively received by young women; however negative responses such as shame, fear, anxiety and depression are more common. The manner in which a girl learns about menstruation and its associated changes may have an impact on her response to the event of menarche. Myth, mystery and superstition have long enveloped the facts about menstruation. In India even mere mention of the topic has been a taboo in the past and even to this date the cultural and social influences appear to be hurdle for advancement of the knowledge of the subject2 . The social practices about menstruation make girl child feel subnormal and may hamper her development. Menarche may remain a traumatic event for her unless she is prepared for it. Adolescent girls constitute a vulnerable group, particularly in India where female child is neglected one. Most studies on female adolescence focus on the gynecological problems but problems of nutritional and psychological origin cannot be ignored1 . Hence present study was undertaken to assess the 1) source of information regarding menstruation, 2) reaction to first and subsequent menstruation, 3) taboos and restrictions as result of menstruation and 4) hygiene practiced during menstruation.

Dolan	2013	A blind spot in girls' education: Menarche and its webs of exclusion in Ghana	Descriptive; Intervention, mixed method	Despite notable progress in girls' education over the last decade, gender-based differences continue to shape educational outcomes. One of the most overlooked of these differences is the process of maturation itself, including menstruation. This paper presents the findings of a study that assessed the impact of sanitary care on the school attendance of post-pubertal girls, as well as the implications of menarche fortheir well-being. The study found that the provision of adequate sanitary care represents a relatively unrecognized but potentially fruitful tool in strategies that aim to improve girls' educational outcomes, one that warrants policy consideration among development planners.
Djalalinia	2012	Parents or School Health Trainers, which of them is Appropriate for Menstrual Health Education?	Intervention, Comparative, mixed method	Objectives:<br>The purpose of this community-based participatory research was to compare different training sources for adolescents' menstrual health education.<br>Methods:<br>From 15 middle schools in Tehran, through quota random sampling, 1823 female students were selected proportionally and allocated randomly to three groups (parent trained, schools' health trainers trained, and control). Following a two-year training program, the adolescents' menstrual health was compared.<br>Results:<br>In the present study, the school health trainers trained group showed a better feeling for menarche, compared to the two other groups ($P < 0.001$). The need for adolescent health training was emphasized by 82% of the participants; they also believed that the appropriate age for such empowerment courses was about 12 years. In the school health trainers trained group, the offered age was significantly lower than in other groups ($P < 0.001$). The adolescents trained by the school health trainers had a better practice of habits related to menstrual and hygiene practices, like having a bath during menstruation and the use of sanitary pads or cotton, compared to their counterpart groups ($P > 0.05$).<br>Conclusion:<br>It is suggested that school-based health training leads to better menstrual health promotion and healthy puberty transition, and school health trainers play a key role in this regard.
Haver (UNICEF)	2013	WASH in Schools Empowers Girls' Education in Masbate Province and Metro Manila, Philippines: An Assessment of Menstrual Hygiene Management in Schools	Qualitative	Emory University, UNICEF Philippines, Plan Philippines and Save the Children Philippines carried out a qualitative assessment of menstruation-related challenges girls face in school. Girls, boys, teachers and mothers at 10 schools in Masbate Province and the National Capital Region were interviewed for their opinions. This report highlights the challenges girls face in school during menses, describes the determinants of these challenges, and outlines the educational and health impacts of these challenges as voiced by the participants.<br>Many girls lack the knowledge, support and resources to manage menstruation in school. Few studies have focused on educational impacts such as concentration, class participation, missed class and absenteeism. This assessment aims to understand the scope of educational and health impacts and challenges across settings in order to foster a broad movement towards mitigating challenges presented by menstruation among adolescent girls. Findings focus on the challenges surrounding menstrual hygiene practices from the participants' perspectives.

Balamurugan, S.S.	2012	Community-based study of reproductive tract infections among women of the reproductive age group in the urban health training centre area in Hubli, Karnataka	Cross-sectional, mixed method	Background: Reproductive tract infections (RTIs) is a global health problem including both sexually transmitted infections (STIs) and non-sexually transmitted infections (non-STIs) of the reproductive tract. RTI/STI is an important concern, as it possess risk for human immunodeficiency virus transmission. Hence a community study was done in Hubli, in terms of active search of the cases based on the symptoms, clinical examination, and feasible laboratory tests along with providing treatment, counselling, and follow-up. Objectives: The objective was to know the prevalence of RTIs among the reproductive age group women and the socio-demographic factors influencing the occurrence of the disease. Materials and Methods: A cross-sectional study was done using a simple random sampling technique to select households. A pretested structured pro forma was used to collect data on RTIs from 656 women of 15-45 years, residing in the field practice area. This was followed by clinical examination and collection of samples for laboratory tests in Urban Health Training Centre, attached to Karnataka Institute of Medical Sciences, Hubli. Results: The prevalence of RTIs among the reproductive age group women was 40.4% based on their symptoms, with majority having abnormal vaginal discharge. The prevalence of RTIs based on clinical finding was 37.4% with majority having vaginitis. The laboratory test revealed a prevalence of 34.3% with majority having Candidiasis. The influence of socio-demographic factors like increased parity, poor socio-economic conditions, poor menstrual hygiene, illiteracy has its direct effect on occurrence of RTI in the community. Conclusion: This depicts that wherever possible, clinical and laboratory findings should support self-reported morbidity to know the exact prevalence of any disease in the community.
Dipali Nemade	2009	Impact of health education on knowledge and practices about menstruation among adolescent school girls of Kalamboli, Navi-mumbai	Cross-sectional, Intervention, Mixed method	This study was undertaken to assess the impact of health education on knowledge regarding menstruation and sources of information, misconceptions, restrictions, status of menstrual hygiene and practices amongst adolescent school girls. A community-based interventional study was conducted among 217 adolescents of Kalamboli, NaviMumbai, Maharashtra; India. A pre-tested questionnaire was administered and later health education regarding menstruation and healthy menstrual practices was imparted to the girls. Post-test was done after 3 months to assess the impact of health education. In the pre-test, menstrual perceptions amongst them were found to be poor and practices incorrect while in the post-test, there was a significant difference in the level of knowledge ($P<0.01$). There was no significant difference in pre and post-test with regard to restrictions followed during menses ($P>0.05$). In the pre-test, it was observed that 51.28 per cent of the girls washed their cloths only with water, 4.27 per cent of them sundried their cloths and 51.28 per cent of them burnt these for final disposal while in the post-test preceding health education, significant improvements were observed in their practices.

Allah	2011	Impact of health education intervention on knowledge and practice about menstruation among female secondary school students in Zagazig city	Intervention, Quasiexperimental	Introduction: The onset of menstruation is part of the maturation process. It is part of the female reproductive cycle that starts when girls become sexually mature at the time of puberty. Menstruation and menstrual practices are still clouded by taboos and socio-cultural restrictions resulting in adolescent girls remaining ignorant of the scientific facts and hygienic health practices, which sometimes result into adverse health outcomes. The aim: This study aimed to improve the impact knowledge level and practices after health education on "menstruation and healthy menstrual practices" among female secondary school students in Zagazig city Research design: A quasiexperimental design was used in the intervention phase of the study to evaluate the impact of health education intervention about menstrual knowledge and hygiene among female secondary school students in Zagazig city. Setting: The study was conducted in one governorate and one private female secondary school in Zagazig city. Subjects: The study comprised of 150 students was chosen from the above mentioned setting. Methods data were collected by using a structure interview questionnaire sheet which covers all items related to menstrual knowledge and hygiene among female secondary school students in Zagazig city. Results: There was a significant improvement in girl's knowledge about nearly all menstruation relevant items in pre-test compared to post-test. In the pre-test period, only 65 (43.33%) girls reported that they wash their genitalia with soap and water whenever they change their cloths/sanitary pads whereas in the post-test period, significant improvement was observed in their menstrual practice ($p<0.01$). Conclusion The present study had revealed unhealthy menstrual practices, low level of knowledge and various misconceptions among adolescent school girls regarding menstruation. The study also clearly pointed out the impact of health education in improving their knowledge and practice. Recommendation: School education program should be imparted to the students. Further, emphasis also needs to be given through workshops and seminars on "Adolescent Reproductive Health".-
Shabnam Omidvar	2010	Factors influencing hygienic practices during menses among girls from south India- A cross sectional study	Cross-sectional, Quantitative	Background: Menstruation is a natural phenomenon among matured females who experience shedding of blood for 1-7 days every month from the age of maturity until menopause. Menstrual hygiene and management is an issue that is insufficiently acknowledged and has not received adequate attention. Aims and Objectives: This study seeks to assess hygienic behavior of unmarried females aged 15 to 22 years and factors affecting their behaviors. Study Design: A cross-sectional study was conducted during 2009-10 on 350 students. They were recruited from educational institutions from a major city in South India. Demographic and menstrual history and hygiene questionnaires were used for obtaining required information. Statistical Packages for the Social Sciences (SPSS) for Windows version 16 was used. Descriptive statistics, Chi-sq and Fisher's exact tests were used for analysis. Results: Mean age of menarche was 13.4±1.2 years; disposable pads were used by two-thirds of the selected girls (68.9%) regardless of age while 45.1% reported to use both disposable and non disposable materials. Frequency of changing pads was 2-3 times a day by 78.3% girls. Socioeconomic Status (SES) of the selected girls and their age influenced choice ofnapkin/pads and other practices such as storage place of napkins; change during night and during school or college hours and personal hygiene. Older girls had better hygienic practices than the younger ones. Seventy six percent of the participants desired for more information regarding menstruation and hygienic practices. Conclusion: A variety of factors are known to affect menstrual behaviors most influential being age and SES. Awareness regarding the need for information about healthy menstrual practices is on rise among

				young women. It is probable that a mechanism be introduced to provide knowledge about menstrual health and self maintenance among women.
Nair	2012	Menstrual Disorders and Menstrual Hygiene Practices in Higher Secondary School Girls	Quantitative	Objective To study the menstrual problems and menstrual hygiene practices of adolescent girls in Thiruvananthapuram City Corporation. Methods Students of class XI and XII in the age group 15-19 years, belonging to ten Higher Secondary Schools within theThiruvananthapuram City Corporation area were selected for the study by multistage sampling procedure and screened using a pretested self evaluation questionnaire. Results Menstrual disorders were reported in 21.1%. The most frequently reported problem during menstruation was dysmenorrhoea (72.4%) followed by oligomenorrhoea (11.3%). Only 11.5% of the girls who had menstrual problems sought treatment and majority from a gynecologist. Out of 81.5% girls who reported vaginal discharge, only 5.7% had abnormal discharge. Menstrual hygiene was adequate in the majority of girls. Conclusions Menstrual disorders are common in adolescence and can have significant consequences on future reproductive health.
Abera	2004	Menarche, Menstruation related Problems and Practices among Adolescent High School Girls in Addis Ababa, 2003/04.	Cross-sectional, mixed method	This is a school-based cross-sectional study conducted among female adolescents who were enrolled for 2003/04 academic year in Addis Ababa Secondary Schools. It was done as part of the curriculum for partial fulfillment of master degree in public health. Pathfinder International and Packard Foundation sponsored it. The study was conducted on randomly selected 863 students. The study looked at research questions such as age at menarche and its correlates; role of parents, schools and friends in the process of maturation; how they are prepared and dealt with menarche, and the current practices; and menstrual hygiene and suitability of school environment during menstruation with its effect on learning process. To reach at the desired objectives, different instruments were used. These were Pre-tested self administered questionnaire; Key informant interview, checklist for observation and focus group discussion. The study revealed that the mean age at menarche was (13.72±1.31) years. The dominant sources of information and advice on menstruation and how to deal with were teachers, mothers, elder sisters and friends. Fathers and brothers were the least consulted. Almost all girls hadn’t expected their menstruation when it happened for the first time. Seventy seven percent of them believed that menstruation was not a female matter which should be kept for oneself, not to talk of it openly to others; 54% of them hadn’t told any body. Girls preferred to get information on menstrual matters from female teachers, mothers, female health personnel friends and elder sisters in their descending order. The most felt needs during early days of menarche were menstrual soak ups and information. Sixty one percent of the girls used rag made soak ups during menarche while the current practice showed that only 52% of them used rag made. Seventy four percent of girls reported to have health problems related to menstruation where abdominal/backache and mood change were the most reported. Absenteeism due to menstruation related health problems was 51%, majority of them for one day when the occurrence of menstruation coincided with week days. vii In Addis Ababa adolescent girls reach at menarche while they are still in elementary schools, before they get sufficient information and counseling on menstruation or how to deal with it. Though most of the girls apparently had classes or obtained information on menstruation related facts that focused more on biologic and hygienic aspect, basically they didn’t address the psychosocial factors. This directly or indirectly may contribute to absenteeism. Besides this, the poor school facilities that couldn’t respond well to the needs of menstruating girls and lack of concern for its management at school are the areas that need attention. Hence, schools should provide the

				minimum acceptable, desirable and affordable standardized menstrual hygiene for girls. Health education or any education related to ARH, maturation process, should focus and complete the parent-student-teacher circle. Intervention programs focusing on school health should work in line with improving this situation as well as take this advantage as a good entry point to addressing other ARH problems including HIV/AIDS.
Pillitteri	2011	School menstrual hygiene management in Malawi: More than toilets	Mixed methods	
WSSCC	2013	Celebrating Womanhood How better menstrual hygiene management is the path to better health, dignity and business	Report; Descriptive	
Asimah	2017	Menstrual hygiene management in Ghana: understanding the socio-cultural, economic, political factors, challenges and opportunities	Exploratory, Qualitative	WaterAid Ghana conducted a study to guide its implementation of a Menstrual Hygiene Management (MHM) Programme in its areas of operation. The study explored existing MHM practices, identifying the endogenous, socio-cultural beliefs, behaviours and practices related to Water Sanitation and Hygiene (WASH) and focussed on the school environment. 319 pupils from 15 schools and 333 households at the community level were engaged across eight Metropolitan, Municipal and District Assemblies. This paper presents some of the key findings of the study
WSSCC	2015	WASH and health for menstrual hygiene management. Training or trainers manual v1.0	Training Manual	
Wateraid	2009	Is menstrual hygiene and management an issue for adolescent school girls? A comparative study of four schools in different settings of Nepal	Cross-sectional, Descriptive, Mixed methods	

M. E. Beksinska, J. Smit, R. Greener, C. S. Todd, M. L. Lee, V. Maphumulo and V. Hoffmann	2015	Acceptability and performance of the menstrual cup in South Africa: a randomized crossover trial comparing the menstrual cup to tampons or sanitary pads	Longitudinal, Intervention, Quantitative	BACKGROUND: In low-income settings, many women and girls face activity restrictions during menses, owing to lack of affordable menstrual products. The menstrual cup (MC) is a nonabsorbent reusable cup that collects menstrual blood. We assessed the acceptability and performance of the MPower(R) MC compared to pads or tampons among women in a low-resource setting. METHODS: We conducted a randomized two-period crossover trial at one site in Durban, South Africa, between January and November 2013. Participants aged 18-45 years with regular menstrual cycles were eligible for inclusion if they had no intention of becoming pregnant, were using an effective contraceptive method, had water from the municipal system as their primary water source, and had no sexually transmitted infections. We used a computer-generated randomization sequence to assign participants to one of two sequences of menstrual product use, with allocation concealed only from the study investigators. Participants used each method over three menstrual cycles (total 6 months) and were interviewed at baseline and monthly follow-up visits. The product acceptability outcome compared product satisfaction question scores using an ordinal logistic regression model with individual random effects. This study is registered on the South African Clinical Trials database: number DOH-27-01134273. RESULTS: Of 124 women assessed, 110 were eligible and randomly assigned to selected menstrual products. One hundred and five women completed all follow-up visits. By comparison to pads/tampons (usual product used), the MC was rated significantly better for comfort, quality, menstrual blood collection, appearance, and preference. Both of these comparative outcome measures, along with likelihood of continued use, recommending the product, and future purchase, increased for the MC over time. CONCLUSION: MC acceptance in a population of novice users, many with limited experience with tampons, indicates that there is a pool of potential users in low-resource settings.
D. U. Ramathuba	2015	Menstrual knowledge and practices of female adolescents in Vhembe district, Limpopo Province, South Africa	Cross-sectional, Descriptive; Exploratory, Quantitative	BACKGROUND: Although sexual issues are openly discussed in the media, sexuality and reproductive functions are treated as taboo. Menstruation is a normal physiologic process, but carries various meanings within cultures and is rarely discussed amongst families and communities. PURPOSE: This study sought to assess the knowledge and practices of secondary school girls towards menstruation in the Thulamela municipality of Limpopo Province, South Africa. METHODS: A quantitative descriptive study design was used and respondents were selected by means of convenience sampling from a population of secondary school girls. The sample consisted of 273 secondary school girls doing Grades 10-12. A self-administered questionnaire was used to collect data, which was analysed by computing frequencies and percentages using the Statistical Package for Social Sciences (SPSS version 12). FINDINGS: The findings revealed that respondents experienced menarche at 13 years and that menstruation is a monthly bleeding (80%) that happens to every female; it is a sign of adulthood (91%). 15% reported that it is the removal of dirt from the stomach and abdomen, 67% indicated the source of menstruation being the uterus, 65% the vagina and 13% from the abdomen. 73% reported having fear and anxiety at the first experience of bleeding and that they could not maintain adequate hygienic practices due to a lack of privacy and sanitary towels. CONCLUSION: Interventions are needed to increase girls' opportunities to discuss menstruation and access information from adults including mothers, parents and guardians. School-based sexuality education should be comprehensive, begin early and be regularly repeated.

E. Anand, J. Singh and S. Unisa	2015	Menstrual hygiene practices and its association with reproductive tract infections and abnormal vaginal discharge among women in India	Cross-sectional, Descriptive; Exploratory, Quantitative	OBJECTIVE: The objective was to explore the determinants of menstrual hygienic practices and its effect on Reproductive Tract Infections (RTI) among ever married women in India. METHODS: District Level Household and Facility Survey-3 (DLHS) India data have been used in the study. The respondents constituted ever married women (N = 577,758) in the age group of 15-49. Bivariate and multivariate techniques were employed using IBM SPSS statistics 20. Individual effects of socio economic, demographic and gynecological factors on menstrual hygienic practices, RTIs and abnormal vaginal discharged respectively were calculated using binary logistic regression. RESULTS: A meager 15% of women used sanitary pad/locally prepared napkins during menstruation in India. Both RTI and Vaginal discharge were positively related with non-use of hygienic methods. The women who used unhygienic method during menstruation were more likely to have any symptom of RTI (OR = 1.046, $p < 0.001$, CI = 1.021-1.071) and vaginal discharge (OR = 1.303, $p < 0.001$, CI = 1.266-1.341). CONCLUSION: The reason for the symptoms of RTI may be diverse and not only limited to the unhygienic menstrual practices although this may be one of the reasons causing reproductive morbidity. Awareness, affordability and privacy are some of the major concerns that need immediate attention to promote the use of sanitary pad during the time of menstruation. Establishing relation between menstrual practices and RTI is in its initial stage of investigation and hence needs further research.
F. Scorgie, J. Foster, J. Stadler, T. Phiri, L. Hoppenjans, H. Rees and N. Muller	2016	Bitten By Shyness: Menstrual Hygiene Management, Sanitation, and the Quest for Privacy in South Africa	Cross-sectional, Descriptive; Qualitative	Little is known about how menstruation is managed in low-income settings and whether existing sanitation systems meet women's needs. Using the 'Photovoice' method with 21 women in participatory workshops and in-depth interviews, we collected data on menstrual hygiene management in three sites in Durban, South Africa. All women reported using disposable sanitary pads. Although they were aware that disposable pads were nonbiodegradable, incompatible with waterborne flush systems, and fill up pit latrines, they had little experience with reusable products. Considerable energy was devoted to concealing and containing 'menstrual waste,' and women expressed concern about inadequate privacy during menstruation. All sites lacked discreet disposal options and reliable water access, while outdoor sanitation facilities were considered unsafe. Findings highlight the need for advocacy to improve safety and privacy of facilities for women in this setting.
S. B. Thakre, S. S. Thakre, S. Ughade and A. D. Thakre	2012	Urban-rural differences in menstrual problems and practices of girl students in Nagpur, India	Cross-sectional, Mixed method	Menstruation in adolescent girls is often associated with menstruation related problems and poor practices. The study was planned to investigate the menstrual related problems and menstrual practices among school going adolescent girls. The study was a community based cross sectional study in a girls school in Nagpur. Majority of menstrual practices were significantly better in urban girls as compared to rural girls ($P<0.05$). Majority of the girls (71.83%) had at least one problem related to menstrual cycles. There was a significant difference in proportion of menstrual problems in rural and urban girls ($P<0.01$). Menstrual problems are a common source of morbidity in this population.

A. Kumar and K. Srivastava	2011	Cultural and social practices regarding menstruation among adolescent girls	Cross-sectional, Descriptive; Quantitative	The study attempts to find out the existing social and cultural practices regarding menstruation, awareness levels, and the behavioral changes that come about in adolescent girls during menstruation, their perception about menarche, how do they treat it, and the various taboos, norms, and cultural practices associated with menarche. The study was conducted on 117 adolescent girls (age 11-20 years) and 41 mothers from various communities and classes in Ranchi comprising residential colonies and urban slums. The findings unfolds many practices: cultural and social restrictions associated with menstruation, myth, and misconception; the adaptability of the adolescent girls toward it; their reaction, reaction of the family; realization of the importance of menstruation; and the changes that have come in their life after menarche and their resistance to such changes. The article also suggests the strategies to improve menstrual health and hygiene among adolescent girls. The study concludes that cultural and social practices regarding menstruation depend on girls' education, attitude, family environment, culture, and belief.
U. M. Lawan, N. W. Yusuf and A. B. Musa	2010	Menstruation and menstrual hygiene amongst adolescent school girls in Kano, Northwestern Nigeria	Cross-sectional, Descriptive; Quantitative	This study examined the knowledge and practices of adolescent school girls in Kano, Nigeria around menstruation and menstrual hygiene. Data was collected quantitatively and analyzed using Epi info version 3.2.05. The mean age of the students was 14.4 +/- 1.2 years; majority was in their mid adolescence. The students attained menarche at 12.9 +/- 0.8 years. Majority had fair knowledge of menstruation, although deficient in specific knowledge areas. Most of them used sanitary pads as absorbent during their last menses; changed menstrual dressings about 1-5 times per day; and three-quarter increased the frequency of bathing. Institutionalizing sexuality education in Nigerian schools; developing and disseminating sensitive adolescent reproductive health massages targeted at both parents and their adolescent children; and improving access of the adolescents to youth friendly services are veritable means of meeting the adolescent reproductive health needs in Nigeria.
E. D. Adinma and J. I. Adinma	2008	Perceptions and practices on menstruation amongst Nigerian secondary school girls	Cross-sectional, Descriptive; Quantitative	This cross-sectional descriptive study was conducted amongst 550 secondary school girls in southeastern Nigeria to determine their perceptions, problems, and practices on menstruation. Majority of the students, (75.6%), were aged 15-17 years. Only 39.3% perceived menstruation to be physiological. Abdominal pain/discomfort, (66.2%), was the commonest medical problem encountered by the respondents, although 45.8% had multiple problems. Medical problems were most commonly discussed with the mother, (47.1%), and least commonly discussed with the teachers, 0.4%. Analgesics, (75.6%), were most commonly used to relieve menstrual pain. Only 10% of respondents used non-pharmacologic remedies. Unsanitary menstrual absorbents were used by 55.7% of the respondents. Menstruation perceptions are poor, and practices often incorrect. A multi-dimensional approach focusing on capacity building of mothers, and teachers on sexuality education skills; using religious organizations as avenues for sexuality education; and effectively using the Mass Media as reproductive health education channels are recommended towards improving adolescents' perceptions and practices on menstruation.

M. L. Schmitt, D. Clatworthy, R. Ratnayake, N. Klaesener-Metzner, E. Roesch, E. Wheeler and M. Sommer	2017	Understanding the menstrual hygiene management challenges facing displaced girls and women: findings from qualitative assessments in Myanmar and Lebanon	Cross-sectional, Descriptive; Exploratory, Quanlitative	BACKGROUND: There is a significant gap in empirical evidence on the menstrual hygiene management (MHM) challenges faced by adolescent girls and women in emergency contexts, and on appropriate humanitarian response approaches to meet their needs in diverse emergency contexts. To begin filling the gap in the evidence, we conducted a study in two diverse contexts (Myanmar and Lebanon), exploring the MHM barriers facing girls and women, and the various relevant sectoral responses being conducted (e.g. water, sanitation and hygiene (WASH), Protection, Health, Education and Camp Management). METHODS: Two qualitative assessments were conducted: one in camps for internally displaced populations in Myanmar, and one with refugees living in informal settlements and host communities in Lebanon. Key informant interviews were conducted with emergency response staff in both sites, and focus group discussion and participatory mapping activities conducted with adolescent girls and women. RESULTS: Key findings included that there was insufficient access to safe and private facilities for MHM coupled with displacement induced shifts in menstrual practices by girls and women. Among staff, there was a narrow interpretation of what an MHM response includes, with a focus on supplies; significant interest in understanding what an improved MHM response would include and acknowledgement of limited existing MHM guidance across various sectors; and insufficient consultation with beneficiaries, related to discomfort asking about menstruation, and limited coordination between sectors. CONCLUSIONS: There is a significant need for improved guidance across all relevant sectors for improving MHM response in emergency context, along with increased evidence on effective approaches for integrating MHM into existing responses.
Wilmouth (PATH)	2012	Interactions between menstrual hygiene management and sanitation systems: Landscape analysis of menstrual hygiene products and a waste-loading model	Modelling	To ensure successful integration of modern menstrual hygiene products (MHPs) with sanitation systems, it is important to understand what is currently done with menstrual waste in urban and peri-urban settings. This is particularly true in light of global trends towards urbanization and the increasing availability of disposable sanitary pads in these settings. The overall Menstrual Management & Sanitation Systems Project is led by the University of Maryland. To support the larger project and address the lack of existing research on women's menstrual hygiene management (MHM), PATH conducted two case studies in South Africa and India from March–December 2012.
Bodat	2013	School absenteeism during menstruation among rural adolescent girls in Pune	Cross-sectional; Quanitative	Background: Menstrual related problems and inadequate school sanitation facilities have an adverse effect on adolescent girl's academic performance and school attendance especially in rural setting. The following study was undertaken to determine school absenteeism during menstruation period. Objective: To assess the impact of menstruation on school atten- dance and factors affecting menstruation management. Methods: This cross sectional study was conducted in rural field practice area of Rural Heath Training Centre under Medical College, Pune among 740 adolescent girls in school by using pre- tested structured-questionnaires. Results: It was noticed that 269 (43.2%) girls used to remain absent from school during menstruation period. School absentee- ism was significantly associated with menstrual disorders, socio economic status, material used during menstruation and abdo- minal pain during menstruation. Nearly 339 (54.5%) of rural girls bring pads/cloths to schools during menstruation while 283 (45.5%) do not bring pads/cloths to schools due to inadequate sanitation facilities. Conclusion: To prevent school absenteeism among these girls and intervention focusing on school menstrual management facilities and reproductive health education sessions is needed.

Khanna	2005	Menstrual Practices and Reproductive Problems: A Study of Adolescent Girls in Rajasthan	Cross-sectional; Qualitative	This article sheds light on some important issues related to menstrual practices and its association with reproductive morbidity among girls. The study indicates that a significantly large proportion of girls were not aware of menstruation when they first experienced it. Mothers, sisters and friends were found to be the major source of information. Much of this information imparted to a young girl is in the form of restrictions on her movements and behaviour. More than three-fourths reported using old cloth during menstruation, and a large proportion of them were reusing the same during subsequent periods. Regression analysis in this study identified schooling, residential status, occupation of father, caste and exposure to media to be the major predictors of safe menstrual practices among adolescent girls in Rajasthan. Importantly, this study found a significantly strong relationship between practices during menstruation and prevalence (reported symptoms) of RTIs. The prevalence of RTIs was more than three times higher among girls having unsafe menstrual practices. The article makes a strong case that ignorance, false perceptions and unsafe practices regarding menstruation are not uncommon among adolescents in the study area, having serious implications for reproductive and sexual health. Further, the study demonstrates that among the determinants for reproductive morbidity, practices during menstruation appear to be the most dominant factor. These findings reinforce the need to bring them out of traditional beliefs, misconceptions and restrictions, and encourage safe and hygienic practices.
Averbach, S.	2009	Duet® for menstrual protection: a feasibility study in Zimbabwe	Intervention; Mixed method	Background: Managing menses is a challenge for women in developing countries. Duet® is a cervical barrier being developed for contraception and STI prevention. We explored the hypothetical acceptability of using Duet as a menstrual cup, among Zimbabwean women. Study Design: A survey and focus group discussions (FGD) were conducted with 43 women aged 18-45 years to gain information about their menstrual practices and attitudes regarding the use of Duet for menstrual protection. Results: All 43 women reported that if Duet were available, they would "definitely" try it, and that it was "very important" that Duet is low cost and easy to clean; 86% reported that using it would make a difference in their lives. FGD findings highlighted unhygienic practices due to the lack of affordable options for menstrual management and a genuine interest in Duet, including its potential use for multiple purposes (contraception, disease prevention and menstrual protection). Conclusions: Accessing affordable and hygienic menstrual protection was a problem for these Zimbabwean women. Duet appeared acceptable and it would be feasible to conduct a user-acceptability study of Duet as a menstrual cup in Zimbabwe. © 2009 Elsevier Inc. All rights reserved.

Oche MO	2012	Menstrual health: The unmet needs of adolescent girls in Sokoto Nigeria	Cross-sectional	The perception and appropriate hygiene practices of girls towards menstruation is closely linked with their level of knowledge and beliefs. This study aimed to assess the level of knowledge on menstruation and hygienic practices among adolescent school girls in an urban city, Nigeria. The study was a cross sectional survey and a total 122 girls from 4 out of the 9 schools' were recruited using a multistage sampling technique to select the schools, and systematic sampling method proportionate to size (proportion of total study unit accounted by each school) after a random selection of the first respondent, using the list of students as the sampling frame and sampling interval of 30. Overall, a total of 79 (65%) of the respondents had high knowledge. 15% of respondents' indicated their major source of information on menstruation from their school teachers. There is a significant gap in knowledge and with minimal role played by the school environment to provide appropriate information during their formative years. The ages of the respondents ($P = 0.93$), education of their mothers ($P = 0.173$) and the sources of information regarding menstruation ($P = 0.575$) were found not to be statistically significant with respect to the knowledge of menstruation while there was a statistically significant relationship between religion ($P = 0.0001$) and level of study of the girls and knowledge of menstruation ($P = 0.048$) Concerning the practice of menstrual hygiene, the majority 106 (87%) of the girls used sanitary pads, only. There was a significant statistically association between education of their mothers ($P = 0.015$), religion ($P = 0.0001$) and occupation of respondents mother ($P = 0.0028$) with respect to the reported menstrual hygiene practices. Hence the need for targeted systematic information to adolescent through curriculum reviews towards better knowledge and practice of menstrual hygiene.
Prateek Bobhate, Saurabh Shrivastava	2011	A Cross Sectional Study of Knowledge and Practices about Reproductive Health among Female Adolescents in an Urban Slum of Mumbai	Cross-sectional, Descriptive; Quantitative	Objective: To study knowledge about reproductive health among female adolescents and to assess their treatment seeking behavior regarding reproductive health problems in an urban slum of Mumbai.<br>Materials and methods: Cross sectional descriptive study was undertaken in an urban slum area of Mumbai for period of 3 months. All adolescent girls from 10 - 19 years of age, who had attained menarche, attending general OPD and STI clinic were included. Subjects were interviewed face to face using pretested semi-structured questionnaire after obtaining informed consent. The questionnaire contained information regarding socio demographic parameters and that related to reproductive health i.e. menstrual hygiene, knowledge and practices related to HIV / AIDS, contraception, abortion, Medical Termination of Pregnancy (MTP), etc. and their treatment seeking behavior in last 3 months for reproductive health problems. Privacy and confidentiality was strictly maintained. Analysis was done with SPSS version 17.<br>Results: Seventy nine (32.8%) subjects had unsatisfactory menstrual hygienic practices. Two hundred twelve (88%) women were aware about availability of ANC services. Sixty six percent of women had correct knowledge of modes of transmission of HIV while only 18.7% knew about safe sexual practices. Education status and early adolescents age group (10 -14 years) was found to be significantly associated with knowledge of adolescents regarding menstruation.<br>Conclusion:Female adolescents should be given appropriate knowledge regarding puberty and sexual health to help them confidently deal with their reproductive and sexual health issues.

Teketo Kassaw Tegegne and Mitike Molla Sisay	2014	Menstrual hygiene management and school absenteeism among female adolescent students in Northeast Ethiopia	Cross-sectional, Mixed methods	Background<br>Adolescence in girls has been recognized as a special period marked with the onset of menarche. Even though menstruation is a natural process, it is associated with misconceptions, malpractices and challenges among girls in developing countries. However, much is not documented; school-absenteeism and dropout are a common problem among girls in rural Ethiopia. Focusing among school girls, this study has examined knowledge about menstruation, determinants of menstrual management and its influence on school-attendance in Northeast Ethiopia.<br>Methods<br>We conducted a mixed-method research combining quantitative and qualitative methods in Northeast Ethiopia. The quantitative study was conducted among 595 randomly selected adolescent school girls. Nine in-depth interviews; five school-dropout girls and four female teachers, and four focus group discussions among school girls were conducted in 2013.<br>Results<br>The mean age at menarche was 13.98 (±1.17) years. About 51% of girls had knowledge about menstruation and its management. Only a third of the girls used sanitary napkins as menstrual absorbent during their last menstruation. Girls from urban areas, had mothers of secondary and above education and, families of higher monthly expenditure had more chance of using sanitary napkins than their counterparts. More than half of the girls reported to have been absent from school during their menstruation period. Those who did not use sanitary napkins were more likely to be absent from school [AOR-95% C.I: 5.37 (3.02 - 9.55)]. Fifty eight percent of girls reported that their school-performance had declined after they had menarche. In addition, the qualitative study indicated that school-dropout was common among girls who experienced teasing and humiliation by classmates when their clothes were stained with blood as they do not use sanitary napkins.<br>Conclusion<br>Though there is an effort to increase girls' school enrollment, lack of basic needs, like sanitary napkins that facilitate routine activates of girls at early adolescence are observed to deter girls' school-attendance in rural Ethiopia. Special support for girl students, especially when they have their first menstruation and separate functioning sanitary facilities are necessities that should be in school at all times if gender equality and girls empowerment is to be achieved.

Table S3. Menstrual hygiene products/absorbents and the materials they are made from in LMIC.

Menstrual Hygiene Product/Absorbent	Material	References (country of study)
**Sanitary Napkins**		
Commercial Disposable Pads	Polymer	(Globally; Sub-Sahara Africa and South Asia focus) George, 2013[37]; (Globally, Training Manual) WSSCC, 2015[38]
Low-cost/ Home-made Pads	Cotton	(Zimbabwe) Averbach et al., 2009[9]; (India) Omidvar and Begum, 2010[23]; (Malawi) Pillitteri, 2011[6]; (Globally; Sub-Sahara Africa and South Asia focus) George, 2013[37]; (Globally, Training Manual) WSSCC, 2015[38]
	Papyrus and waste paper pulp e.g. Makapads	(Developing Countries) Tilley et al., 2013[39]; (Uganda) Crofts and Fisher, 2012[7]
	Poly cotton e.g. Afripads	(Uganda) Crofts and Fisher, 2012[7]
	Banana leaves	(Developing Countries) Tilley et al., 2013[39]
	Gauze and cotton e.g. BRAC pads	(Developing Countries) Bharadwaj and Patkar, 2004[40]; (Egypt) El-Gilany et al., 2005[1]; (Developing Countries) Tilley et al., 2013[39]
	Cloth pad filled with ash/sand/dry leaves	(Developing Countries) Bharadwaj and Patkar, 2004[40]; (Globally, Training Manual) WSSCC, 2015[38]
**Traditional Materials**		
Cloth	Cotton	(India) Baridalyne and Reddaiah, 2004[19]; (India) Nemade et al., 2009[22]; (Pakistan) Ali and Rizvi, 2010[34]; (India) Omidvar and Begum, 2010[23]; (Uganda) Crofts and Fisher, 2012[7]; (Iran) Djalalinia et al., 2012[36]; (India) Kamaljit et al., 2012[27]; (India) Khanna et al., 2005[21]; (LMIC) Sumpter and Torondel, 2013[41]; (LMIC) Chandra-Mouli and Patel, 2017[42]; (Resource Poor Countries) Kuhlmann et al., 2017[43]
	Wool	(India) Ali and Rizvi, 2010[34]; (Resource Poor Countries) Kuhlmann et al., 2017[43]
	Nylon	(India) Nemade et al., 2009[22]; (Egypt) Allah and Elsabagh, 2011[2]
	Terrycloth	(India) Nemade et al., 2009[22]

Clothing	Sari, dupattas, shalwar, skirts	(Nepal) WaterAid, 2009[33]; (India) Ali and Rizvi, 2010[34]; (Globally; Sub-Sahara Africa and South Asia focus) George, 2013[37]
	Underwear	(LMIC) Sumpter and Torondel, 2013[41]; (Ethiopia) Tegegne et al., 2014[4]; (India) van Eijik et al., 2016[44]
Other Absorbents	Toilet paper/Tissue	(Zimbabwe) Averbach et al., 2009[9]; (Uganda) Crofts and Fisher, 2012[7]; (Nigeria) Oche et al., 2012[13]; (LIC) Sommer et al., 2013[45]; (LMIC) Sumpter and Torondel, 2013[41]; (Globally; Sub-Sahara Africa and South Asia focus) George, 2013[37]; (Nigeria) Aluko et al., 2014[12]; (Zambia) Lahme et al., 2016[8]; (South Africa) Scorgie et al., 2016[16]; (Ethiopia) Wall et al., 2016[5]; (LMIC) Chandra-Mouli and Patel, 2017[42]; (Resource Poor Countries) Kuhlmann et al., 2017[43]
	Cotton wool	(Zimbabwe) Averbach et al., 2009[9]; (Pakistan) Ali and Rizvi, 2010[34]; (Uganda) Crofts and Fisher, 2012[7]; (LMIC) Sumpter and Torondel, 2013[41]; (Resource Poor Countries) Kuhlmann et al., 2017[43]
	Newspaper	(Zimbabwe) Averbach et al., 2009[9]; (Uganda) Crofts and Fisher, 2012[7]; (Globally; Sub-Sahara Africa and South Asia focus) George, 2013[37]; (South Africa) Ramathuba et al., 2015[15]
	Hand towels	(South Africa) Ramathuba et al., 2015[15]
	Sponge	(LMIC) Sumpter and Torondel, 2013[41]; (Ethiopia) Wall et al., 2016[5]
	Dry leaves, grass	(Uganda) Crofts and Fisher, 2012[7]; (Globally, Training Manual) WSSCC, 2015[38]; (Resource Poor Countries)Kuhlmann et al., 2017[43]
	Straw	(Globally; Sub-Sahara Africa and South Asia focus) George, 2013[37]
	Ash	(Globally; Sub-Sahara Africa and South Asia focus) George, 2013[37]
**Other Menstrual Products/Absorbents**		
Commercial Products	Menstrual Cups e.g. Polyurethane "Duets" or Silicone "Moon cups" "MPower"	(Developing Countries) Tilley et al., 2013[39]; (South Africa) Beksinska et al., 2015[14]

Tampons	(Zimbabwe) Averbach et al., 2009[9]; (Uganda) Crofts and Fisher, 2012[7]; (South Africa) Beksinska et al., 2015[14]

Table S4. Disposal practices reported in studies.

First author, year	Country	Setting	N	Disposal method practiced (%)					Comments
				Throwing in the open	Throwing in latrine	Burning	Burying	Dustbin/ routine waste	
Haque, 2014[17]	Bangladesh	School	416	6.2	11.8			82.0	Figures represent endline findings post intervention. findings for disposal by burning, burying, dustbin were combined, and considered as appropriate disposal in this study
El-Gilany, 2005[1]	Egypt	School	664			2.5	0.8	96.7	Cloth use was much higher than sanitary pad use in this study
Allah, 2011[2]	Egypt	School	102	33.3<br>22.5		10.8<br>4.9	2.9<br>0.9	52.9<br>71.6	Pre-intervention (disposal related to cloth use)<br>Post intervention (disposal related to cloth use)
Gultie, 2014[46]	Ethiopia	school	492	6.3	69.3		0.4	14.4	
Tegegne 2014[4]	Ethiopia	School		33.4	77.5			3.5	
Asimah, 2017[10]	Ghana	school	319	19	34			27	
Thakre, 2011[26], 2012[47]	India	School			7.9	46.9		45.2	Urban figures are reported in this table. Rural-urban differences were noted with urban population more likely that rural population to throw used absorbents with routine household waste
Van Eijk et al 2016[44]	India	Mixed	138 studies 97070 girls	15.00<br>30.00	7.00	23.00	12.00	70.00<br>51.00	Urban pooled proportion<br>Slum pooled proportion

			117	12.8		51.3	3.4	32.5	Pre-intervention (disposal related to cloth use)
				2.6		24.8	0.9	71.8	Post intervention (disposal related to cloth use)
Nemade, 2009[22]	India	School	166	3.0	0.6			96.4	Pre-intervention (disposal related to pad use)
				0.6	0.0			99.4	Post intervention (disposal related to pad use)
				5.3	0.9			93.9	Pre-intervention (disposal related to pad use)
				0.9	0.0			99.1	Post intervention (disposal related to pad use)
Nair, 2012[28]	India	school	3443	2.7	7.9	76	11		Regarding the disposal of cloth
Bobhate, 2011[24]	India	Community	241	14.5				54.8	Method of disposal for both pads and cloth
WaterAid, 2009[33]	Nepal	School	136			19	43	38	
Aniebue 2009[48]	Nigeria	School	273/222	5	34.1	35.5		27.8	Disposal of menstrual absorbents by trained respondents
				13	48.7	25.2		18.5	Disposal of menstrual absorbents by untrained respondents
Oche, 2012[13]	Nigeria	Schools	122			53			41% of pads were wrapped and thrown away (but the article does not provide clarity on whether these pads were thrown in the open or along with routine waste
Beksinska, 2015[14]	South Africa	Community	110		6.4			87.5	Menstrual cup trial

Table S5. Overview of different incinerator technologies including type of use, advantages, disadvantaged and examples of current use. The table has been adapted from PATH (2017) [49].

Incinerator type	Type of use	Advantages	Disadvantages	Examples of use
Clay pots (Matka)	Household	• Low cost<br>• Easily available<br>• Easy to use<br>• Use locally available fuel	• No emission control<br>• Burn at low temperature, hence waste may not burn efficiently<br>• May not work for pads with high moisture content and high content of super absorbent polymers<br>• Ash might not be safe to use for gardening<br>• Design is highly variable and not standardised	• Households Papna Mau village, Uttar Pradesh, India[50]
Low cost locally made incinerators	Institutional/public settings, households	• Low cost<br>• Easy to install/build from local available materials<br>• Men can be involved in building incinerators, hence reducing taboos<br>• Easy to use and maintain<br>• Use locally available fuel	• No emission control<br>• Burn at low temperature, hence waste may not burn efficiently<br>• May not work for pads with high moisture content and high content of super absorbent polymers<br>• Ash might not be safe to use for gardening<br>• Design is highly variable and not standardised	• Schools in Nepal[51]<br>• Schools in Uttar Pradesh, India[52]<br>• Rural schools in Tamil Nadu[53]<br>• Urban school Malawi[6]<br>• Household, Tamil Nadu, India[54]<br>• Communal EcoSan toilet, Tamil Nadu, India[54]

Electric incinerators	Institutional/public settings	• More expensive models have emission control features such as filters<br>• No need for fuel<br>• Some models have quality certification	• Dependent on electric supply<br>• High cost<br>• Unclear if they can efficiently burn high moisture content and pads with high content of super absorbent polymers<br>• Variation in design<br>• No standard quality certification	• Public toilet, Chennai Central railway station[55]<br>• Public toilet, Dindigul bus stand, Tamil Nadu, India[56]<br>• Schools, Tamil Nadu, India[57]<br>• Schools, Kerala, Delhi, Rajasthan, Madhya Pradesh, Maharashtra, Haryana and West Bengal, India[58]
High temperature incinerators for bio-medical waste	Centralised incineration	• Waste burned at high temperatures (over 800C)<br>• Can incinerate all types of pads	• Collection, transportation and storage of segregated menstrual waste is required to a central treatment facility<br>• Classification of MHM waste as bio-medical waste would be needed	
Incinerators with waste to energy technology	Institutional/public settings	• Produce energy from waste<br>• Developed to be highly controlled systems with emission control<br>• Developed to cope with all types of pads	• Few incinerators exist today<br>• High cost<br>• More development needed	

Table S6. Authors' recommendations

Recommendations
Research to inform policy, practice and technology development
• Public and environmental health research confirming risks of pathogen transmission from waste products disposed in the open or handled by waste or sanitation workers<br>• Product research on composition and quality of disposable, compostable and reusable menstrual hygiene products, and health effects of these products linked with additives and hygienic use (especially duration of use)<br>• Research to estimate the health and environmental effects of waste management strategies (e.g., incineration, composting) on different types of products (e.g., emissions from incinerating different types of sanitary pads)<br>• Further research and development on thermal treatment strategies and technology, examining incinerator types being used, their installation and maintenance, temperatures of operation and emissions from incinerators<br>• In-country or country-specific operational research on use of incinerators for menstrual waste management in public toilets and institutional settings (particularly schools and workplaces where installations are common)<br>• Policy research on governments' positioning of menstrual waste management, procurement processes and budgetary allocations for menstrual waste solutions
Comprehensive MHM programs to address product use and menstrual waste disposal practices in public settings
• MHM programs to include attention to informed product choice (whereby girls and women are informed of the range of menstrual hygiene products available, their hygienic use, and their advantages and disadvantages), as well as information on and solutions for the safe management of menstrual waste in community, institutional, and public settings. Innovative yet simple behavior strategies to promote appropriate disposal practices to be identified, tested and scaled up<br>• Attention to operations and maintenance of waste management solutions, especially incinerator technologies in different settings, with appropriate and detailed training and guidance to operators and institutions to ensure smooth and efficient functioning of technologies<br>• Clear articulation of and operational guidance on menstrual waste management from the time of segregation and disposal by user, to collection, transportation, and final treatment and disposal of waste products

• Implementation research and monitoring indicators for interventions to include attention to menstrual waste management practices and solutions<br>• The unique menstrual hygiene needs of girls and women who are differently abled or in vulnerable situations (e.g. camps, disaster settings) including their needs and challenges with disposal to be understood and considered within the scope of both WASH and MHM programs in general, including waste management strategies<br>• WASH and MHM sector actors to create global and national platforms for cross-learning and knowledge sharing on the evolving menstrual hygiene product landscape and waste management solutions, engaging industry, government and non-governmental stakeholders
Policy advocacy for safe and appropriate management of menstrual waste
• Menstrual waste management to be positioned as a critical issue relevant for all government departments addressing MHM in various ways (through education, health, sanitation, women's empowerment / gender equality), with guidance on how government departments can coordinate to aid comprehensive programming on MHM<br>• Government to develop and institute performance standards and regulatory and enforcement mechanisms for menstrual hygiene products and waste management solutions and technologies, and introduce regular monitoring to ensure quality and adherence to standards.<br>• Adequate budgetary allocations through government policies and schemes for all components of MHM, including menstrual hygiene products and waste management solutions<br>• Global guidance documents to include greater attention to MHM to include waste management. For instance, documentation from WHO or UNICEF on core questions and indicators for monitoring WASH in schools and the SDGs should include further guidance on MHM and waste management as part of the service ladders<br>• Global and national platforms (e.g. conferences, networks) for advocacy on sanitation and MHM to include attention to menstrual waste management particularly through research, technology development and intervention implementation

Reference

1. El-Gilany, A.H.; Badawi, K.; El-Fedawy, S. Menstrual hygiene among adolescent schoolgirls in Mansoura, Egypt. *Reprod. Health Matters* **2005**, *13*, 147–152, doi:10.1016/s0968-8080(05)26191-8.
2. Allah, E.A.E.E. Impact of Health Education Intervention on Knowledge and Practice about Menstruation among Female Secondary School Students in Zagazig City. *J. Am. Sci.* **2011**, *7*, 737–747.
3. Abera, Y. *Menarche, Menstruation Related Problems and Practices among Adolescent High School Girls in Addis Ababa, 2003/04*; Addis Ababa University: Addis Ababa, Ethiopia, 2004.
4. Tegegne, T.K.; Sisay, M.M. Menstrual hygiene management and school absenteeism among female adolescent students in Northeast Ethiopia. *BMC Public Health* **2014**, *14*, 1118.
5. Wall, L.L.; Belay, S.; Bayray, A.; Salih, S.; Gabrehiwot, M. A community-based study of menstrual beliefs in Tigray, Ethiopia. *Int. J. Gynaecol. Obstet.* **2016**, *135*, 310–313, doi:10.1016/j.ijgo.2016.05.015.
6. Pillitteri, S.P. *School Menstrual Hygiene Management in Malawi: More than Toilets*; WaterAid: London, UK, 2011.
7. Crofts, T.; Fisher, J. Menstrual hygiene in Ugandan schools: An investigation of low-cost sanitary pads. *J. Wate Sanit. Hyg. Dev.* **2012**, *2*, 50–58, doi:10.2166/washdev.2012.067.
8. Lahme, A.M.; Stern, R.; Cooper, D. Factors impacting on menstrual hygiene and their implications for health promotion. *Glob. Health Promot.* **2016**, *2016*, doi:10.1177/1757975916648301.
9. Averbach, S.; Sahin-Hodoglugil, N.; Musara, P.; Chipato, T.; van der Straten, A. Duet for menstrual protection: A feasibility study in Zimbabwe. *Contraception* **2009**, *79*, 463–468.
10. Asimah, S.A.; Diabene, P.Y.; Wellington, S.N.L. Menstrual hygiene management in Ghana: Understanding the socio-cultural, economic, political factors, challenges and opportunities. In Proceedings of the 40th WEDC International Conference, Loughborough, UK, 24–28 July 2017.
11. Adinma, E.D.; Adinma, J.I. Perceptions and practices on menstruation amongst Nigerian secondary school girls. *Afr. J. Reprod. Health* **2008**, *12*, 74–83.
12. Aluko, O.O.; Oluya, O.M.; Olaleye, O.A.; Olajuyin, A.A.; Olabintan, T.F.; Oloruntoba-Oju, O.I. Knowledge and menstrual hygiene practices among adolescents in senior secondary schools in Ile Ife, south-western Nigeria. *J. Wate Sanit. Hyg. Dev.* **2014**, *4*, 248–256, doi:10.2166/washdev.2014.084.
13. Oche, M.O.; Umar, A.S.; Gana, G.J.; Ango, J.T. Menstrual health: The unmet needs of adolescent girls in Sokoto, Nigeria. *Sci. Res. Essays* **2012**, *7*, 410–418.
14. Beksinska, M.E.; Smit, J.; Greener, R.; Todd, C.S.; Lee, M.L.; Maphumulo, V.; Hoffmann, V. Acceptability and performance of the menstrual cup in South Africa: A randomized crossover trial comparing the menstrual cup to tampons or sanitary pads. *J. Women's Health* **2015**, *24*, 151–158, doi:10.1089/jwh.2014.5021.
15. Ramathuba, D.U. Menstrual knowledge and practices of female adolescents in Vhembe district, Limpopo Province, South Africa. *Curationis* **2015**, *38*, doi:10.4102/curationis.v38i1.1551.

16. Scorgie, F.; Foster, J.; Stadler, J.; Phiri, T.; Hoppenjans, L.; Rees, H.; Muller, N. "Bitten by Shyness": Menstrual Hygiene Management, Sanitation, and the Quest for Privacy in South Africa. *Med. Anthropol.* **2016**, *35*, 161–176, doi:10.1080/01459740.2015.1094067.
17. Haque, S.E.; Rahman, M.; Itsuko, K.; Mutahara, M.; Sakisaka, K. The effect of a school-based educational intervention on menstrual health: An intervention study among adolescent girls in Bangladesh. *BMJ Open* **2014**, *4*, e004607, doi:10.1136/bmjopen-2013-004607.
18. Balamurugan, S.S.; Bendigeri, N.D. Community-based study of reproductive tract infections among women of the reproductive age group in the urban health training centre area in Hubli, Karnataka. *Indian J. Community Med.* **2012**, *37*, 34–38.
19. Baridalyne, N.; Reddaiah, V.P. Menstruation: Knowledge, beliefs and practices of women in the reproductive age group residing in an urban resettlement colony of Delhi. *Health Popul.* **2004**, *27*, 9–16.
20. Deo, D.S.; Ghattargi, C.H. Perceptions and Practices Regarding Menstruation: A Comparative Study in Urban and Rural Adolescent Girls. *Indian J. Community Med.* **2005**, *30*, 33–34.
21. Khanna, A.; Goyal, R.S.; Bhawsar, R. Menstrual Practices and Reproductive Problems: A Study of Adolescent Girls in Rajasthan. *J. Health Manag.* **2005**, *7*, 91–106.
22. Nemade, D.; Anjenaya, S.; Gujar, R. Impact of health education on knowledge and practices about menstruation among adolescent school girls of Kalamboli, Navi-Mumbai. *Health Popul.* **2009**, *32*, 167–175.
23. Omidvar, S.A.B.K. Factors influencing hygienic practices during menses among girls from south India—A cross sectional study. *Int. J. Collab. Res. Internal Med. Public Health* **2010**, *2*, 411–423.
24. Bobhate, P.S.; Saurabh, M.D.; Shrivastava, M.D. A Cross Sectional Study of Knowledge and Practices about Reproductive Health among Female Adolescents in an Urban Slum of Mumbai. *J. Fam. Reprod. Health* **2011**, *5*, 117–124.
25. Kumar, A.; Srivastava, K. Cultural and social practices regarding menstruation among adolescent girls. *Soc. Work Public Health* **2011**, *26*, 594–604, doi:10.1080/19371918.2010.525144.
26. Thakre, S.B.; Thakre, S.S.; Reddy, M.; Rathi, N.; Pathak, K.; Ughade, S. Menstrual hygiene: Knowledge and practice among adolescent school girls of Saoner, Nagpur District. *J. Clin. Diagn. Res.* **2011**, *5*, 1027–1033.
27. Kamaljit, K.; Balwinder, A.; Gurmeet, K.S.; Neki, N.S. Social beliefs and practices associated with menstrual hygiene among adolescent girls of Amritsar, Punjab, India. *J. Int. Med. Sci. Acad.* **2012**, *25*, 69–70.
28. Nair, M.K.C.; Chacko, D.S.; Darwin, M.R.; Padma, K.; George, B.; Russel, P.S. Menstrual Disorders and Menstrual Hygiene Practices in Higher Secondary School Girls. *Indian J. Pediatr.* **2012**, *79*, S74–S78.
29. Bodat, S.; Ghate, M.M.; Majumdar, J.R. School Absenteeism During Menstruation Among Rural Adolescent Girls in Pune. *Natl. J. Community Med.* **2013**, *4*, 212–216.

30. Thakur, H.; Aronsson, A.; Bansode, S.; Stalsby Lundborg, C.; Dalvie, S.; Faxelid, E. Knowledge, Practices, and Restrictions Related to Menstruation among Young Women from Low Socioeconomic Community in Mumbai, India. *Front. Public Health* **2014**, *2*, 72, doi:10.3389/fpubh.2014.00072.
31. Anand, E.; Singh, J.; Unisa, S. Menstrual hygiene practices and its association with reproductive tract infections and abnormal vaginal discharge among women in India. *Sex. Reprod. Healthc.* **2015**, *6*, 249–254, doi:10.1016/j.srhc.2015.06.001.
32. Mishra, S.K.; Dasgupta, D.; Ray, S. A study on the relationship of sociocultural characteristics, menstrual hygiene practices and gynaecological problems among adolescent girls in Eastern India. *Int. J. Adolesc. Med. Health* **2016**, *29*, doi:10.1515/ijamh-2015-0111.
33. Nepal, W. *Is Menstrual Hygiene and Management an Issue for Adolescent School Girls? A Comparative Sutdy of Four Schools in Different Settings of Nepal*; Wateraid: Kathmandu, Nepal, 2009.
34. Ali, T.S.; Rizvi, S.N. Menstrual knowledge and practices of female adolescents in urban Karachi, Pakistan. *J. Adolesc.* **2010**, *33*, 531–541, doi:10.1016/j.adolescence.2009.05.013.
35. Haver, J.; Caruso, B.; Ellis, A.; Villasenor, J.; Andes, K.; Freeman, M. *WASH in Schools Empowers Girls' Education in Masbate Province and Metro Manila, Philippines: An Assessment of Menstrual Hygiene Management in Schools*; United Nations Children's Fund: New York, NY, USA, 2013.
36. Djalalinia, S.; Tehrani, F.R.; Afzali, H.M.; Hejazi, F.; Peykari, N. Parents of School Health Trainers, Which of them is Appropriate for Menstrual Health Education? *Int. J. Prev. Med.* **2012**, *3*, 622–627.
37. George, R. *Celebrating Womanhood: How Better Menstrual Hygiene Management is the Path to Better Health, Dignity and Business*; WSSCC (Water Supply & Sanitation Collaborative Council): London, UK, 2013.
38. Council WWSSC. *WASH and Health for Menstrual Hygiene Management Training of Trainer Manual v1.0*; Council WWSSC: Geneva, Switzerland, 2015.
39. Tilley, E.; Bieri, S.; Kohler, P. Sanitation in developing countries: A review through a gender lens. *J. Wate Sanit. Hyg. Dev.* **2013**, *3*, 298–314, doi:10.2166/washdev.2013.090.
40. Bharadwaj, S.; Patkar, A. Menstrual hygiene and management in developing countries: Taking stock. *Junction Soc.* **2004**, *3*, 20.
41. Sumpter, C.; Torondel, B. A systematic review of the health and social effects of menstrual hygiene management. *PLoS ONE* **2013**, *8*, e62004, doi:10.1371/journal.pone.0062004.
42. Chandra-Mouli, V.; Patel, S.V. Mapping the knowledge and understanding of menarche, menstrual hygiene and menstrual health among adolescent girls in low- and middle-income countries. *Reprod. Health* **2017**, *14*, 30, doi:10.1186/s12978-017-0293-6.
43. Kuhlmann, A.S.; Henry, K.; Wall, L.L. Menstrual Hygiene Management in Resource-Poor Countries. *Obstet. Gynecol. Surv.* **2017**, *72*, 356–376, doi:10.1097/ogx.0000000000000443.
44. van Eijk, A.M.; Sivakami, M.; Thakkar, M.B.; Bauman, A.; Laserson, K.F.; Coates, S.; Phillips-Howard, P.A. Menstrual hygiene management among adolescent girls in India: A systematic review and meta-analysis. *BMJ Open* **2016**, *6*, e010290, doi:10.1136/bmjopen-2015-010290.
45. Sommer, M.; Kjellen, M.; Pensulo, C. Girls' and women's unmet needs for menstrual hygiene management (MHM): The interactions between MHM and sanitation systems in low-income countries. *J. Wate Sanit. Hyg. Dev.* **2013**, *3*, 283–297, doi:10.2166/washdev.2013.101.

46. Gultie, T.; Hailu, D.; Workineh, Y. Age of Menarche and Knowledge about Menstrual Hygiene Management among Adolescent School Girls in Amhara Province, Ethiopia: Implication to Health Care Workers and School Teachers. *PLoS ONE* **2014**, *9*, e108644.
47. Thakre, S.B.; Thakre, S.S.; Ughade, S.; Thakre, A.D. Urban-rural differences in menstrual problems and practices of girl students in Nagpur, India. *Indian Pediatr.* **2012**, *49*, 733–736.
48. Aniebue, U.U.; Aniebue, P.N.; Nwankwo, T.O. The impact of pre-menarcheal training on menstrual practices and hygiene of Nigerian school girls. *Pan Afr. Med. J.* **2009**, *2*, 9.
49. PATH. *Management of Menstrual Waste*; PATH: Seattle, WA, USA, 2017.
50. Correia, R. Learn How a Village in UP is Cleaning up Menstrual Waste Sustainable—With a Home-Based Solution! In *TBI Blogs*; LinkedIn: Mountain View, CA, USA, 2017.
51. Elawati, K.C. *A Study of Menstrual Hygiene and Impact of Pad Incinerator: A Comparative Study in Bharatpur, Guleria, Tikapur and Butwal*; Environment & Public Health Organization: Kathmandu, Nepal, 2012.
52. Tripathy, A. Menstrual hygiene: Engaging with governments to strengthen programmes of action. In Proceedings of the 39th WEDC International Conference, Kumasi, Ghana, 11–15 July 2016.
53. India, G.O. *Incinerator for School Toilet Waste Case Study: Tamil Nadu*; Rajiv Gandhi National Drinking Water Supply, Department of Drinking Water Supply, Ministry of Rural Development: Tamil Nadu, India, 2007.
54. PATH. *Menstrual Management and Sanitation Systems: Findings from Two Case Studies in South Africa and India*; PATH: Seattle, WA, USA, 2013.
55. Malhotra, N. IITian's Gift to Women at Chennai Central Station Is a Big Win for Menstrual Hygiene. *Better India*, **2016**.
56. Reporter, S. Now, women-friendly toilets in Dindigul bus stand. Available online: https://www.thehindu.com/todays-paper/tp-national/tp-tamilnadu/now-women-friendly-toilets-in-dindigul-bus-stand/article21286438.ece (accessed on 16 August 2018).
57. Reporter, S. Eco-friendly napkin incinerators given to 25 schools and colleges. Available onlie: https://www.thehindu.com/news/cities/Tiruchirapalli/ecofriendly-napkin-incinerators-given-to-25-schools-and-colleges/article4742351.ece (accessed on 16 August 2018).
58. IANS. Sanitary napkin vending machines and incinerators in 150 Kerala schools. Available onlie: https://www.thenewsminute.com/article/sanitary-napkin-vending-machines-and-incinerators-150-kerala-schools-53218 (accessed on 16 August 2018)

© 2018 by the authors. Submitted for possible open access publication under the terms and conditions of the Creative Commons Attribution (CC BY) license (http://creativecommons.org/licenses/by/4.0/).
